# Advances in the Understanding of Skin Cancer: Ultraviolet Radiation, Mutations, and Antisense Oligonucleotides as Anticancer Drugs

**DOI:** 10.3390/molecules24081516

**Published:** 2019-04-17

**Authors:** Kateryna V. Laikova, Volodymyr V. Oberemok, Alisa M. Krasnodubets, Nikita V. Gal’chinsky, Refat Z. Useinov, Ilya A. Novikov, Zenure Z. Temirova, Mikhail V. Gorlov, Nikita A. Shved, Vadim V. Kumeiko, Tatiana P. Makalish, Evgeniya Y. Bessalova, Iryna I. Fomochkina, Andrey S. Esin, Mikhail E. Volkov, Anatoly V. Kubyshkin

**Affiliations:** 1 Medical Academy named after S.I. Georgievsky, V.I. Vernadsky Crimean Federal University, Lenin Boulevard 5/7, 295051 Simferopol, Crimea; botan_icus@mail.ru (K.V.L.); wwwzzznnn333@gmail.com (Z.Z.T.); gemini_m@list.ru (T.P.M.); evgu79@mail.ru (E.Y.B.); fomochkina_i@mail.ru (I.I.F.); kubyshkin_av@mail.ru (A.V.K.); 2 Research Institute of Agriculture of Crimea, Kiyevskaya St. 150, 295493, Simferopol, Crimea; 3 Taurida Academy, V.I. Vernadsky Crimean Federal University, Vernadsky Av. 4, 295007 Simferopol, Crimea; genepcr@mail.ru (V.V.O.); pcr.product@gmail.com (N.V.G.); useinovrefat@gmail.com (R.Z.U.); i.nowikow2012@mail.ru (I.A.N.); 4 Nikita Botanical Gardens—National Scientific Centre RAS, Nikitsky spusk 52, vil. Nikita, 298648 Yalta, Crimea; 5 D. Mendeleev University of Chemical Technology of Russia, Miusskaya sq. 9, 125047 Moscow, Russia; mikgorlov@gmail.com (M.V.G.); sin.sad92@gmail.com (A.S.E.); 6 Centre for Genomic and Regenerative Medicine, School of Biomedicine, Far Eastern Federal University, Sukhanova St. 8, 690090 Vladivostok, Russia; nikitawayfarer@yandex.ru (N.A.S.); vkumeiko@yandex.ru (V.V.K.); 7 National Scientific Center of Marine Biology, Far Eastern Branch of Russian Academy of Sciences, Palchevsky St. 17, 690041 Vladivostok, Russia; 8 Ltd “NPF Syntol”, Timiryazevskaya St. 42, 127434 Moscow, Russia; mikki1@mail.ru

**Keywords:** melanoma, basal cell carcinoma, squamous cell carcinoma, Merkel cell carcinoma, dermatofibrosarcoma protuberans, ultraviolet radiation, mutations, skin, antisense oligonucleotides

## Abstract

Skin cancer has always been and remains the leader among all tumors in terms of occurrence. One of the main factors responsible for skin cancer, natural and artificial UV radiation, causes the mutations that transform healthy cells into cancer cells. These mutations inactivate apoptosis, an event required to avoid the malignant transformation of healthy cells. Among these deadliest of cancers, melanoma and its ‘younger sister’, Merkel cell carcinoma, are the most lethal. The heavy toll of skin cancers stems from their rapid progression and the fact that they metastasize easily. Added to this is the difficulty in determining reliable margins when excising tumors and the lack of effective chemotherapy. Possibly the biggest problem posed by skin cancer is reliably detecting the extent to which cancer cells have spread throughout the body. The initial tumor is visible and can be removed, whereas metastases are invisible to the naked eye and much harder to eliminate. In our opinion, antisense oligonucleotides, which can be used in the form of targeted ointments, provide real hope as a treatment that will eliminate cancer cells near the tumor focus both before and after surgery.

## 1. Introduction

**Figure 1:**
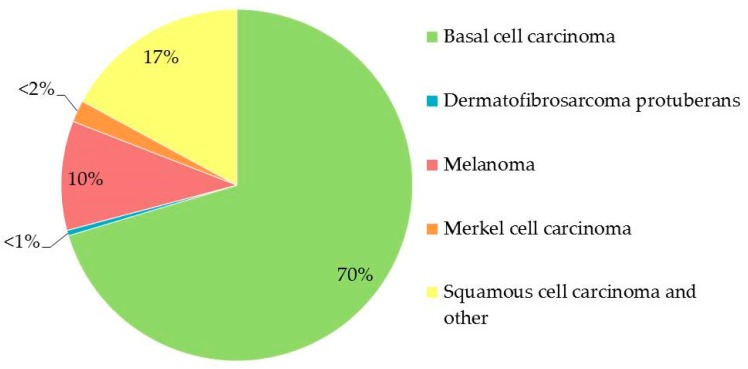
Incidence of different types of skin cancer.

Skin cancer is the most common malignancy in the world, affecting men and women of every skin color [1]. The incidence of cancers of all kinds is increasing; among newly diagnosed cancers, one in three is a skin cancer [2] (Figure 1). Individuals in white- or light-skinned populations bear the brunt of skin cancer; the most common cancers in these populations are melanoma and non-melanoma skin cancer (including squamous cell carcinoma, basal cell carcinoma, Bowen’s disease, keratoacanthoma, and actinic keratosis). Huge amounts of time and money are spent on research worldwide in an effort to address cancer; as a result, the associated mortality rates are stable and possibly decreasing [3]. That said, the World Health Organization still estimates that, each year, between 2 and 3 million non-melanoma skin cancers and ~132,000 melanomas occur globally [4]. The progression and general characteristics of melanomas are far more severe than those of non-melanoma skin cancers; the incidence of the latter varies widely, with the highest rates reported in Australia [5] and the lowest rates in parts of Africa [6]. This pattern results from both skin color and exposure to sunlight in these areas. With an incidence of ~80%, basal cell carcinomas comprise the bulk of non-melanoma skin cancers [7]. Basal cell and squamous cell skin cancers are easier to treat and grow more slowly than melanomas, which is good, as they occur more often. On the other hand, melanoma, which is highly metastatic, drug-resistant, and aggressive, is responsible for 75% of all deaths from skin cancer, even though it comprises only 5–10% of the cases diagnosed. The burden imposed by melanoma is borne primarily by Australasian, North American, and European populations, as well as individuals from all populations who are elderly and/or male [8].

Two unusual skin cancers are Merkel cell carcinoma and dermatofibrosarcoma protuberans, both of which occur rarely. While the incidence of Merkel cell carcinoma is ~4.5- to 5.5-fold lower than that of melanoma, it is particularly aggressive and metastasizes at an early stage. Within 2 years of diagnosis, it has a relative mortality of approximately 30%, which reaches 50% within 5 years of diagnosis [9]. Merkel cell carcinoma is the second leading cause of skin cancer death after melanoma, even though it is responsible for less than 1% of malignant tumors of the skin [10]. Dermatofibrosarcoma protuberans, which occurs ~1.3–7.5 times less often than even Merkel cell carcinoma, rarely metastasizes [11] and in general has a much better prognosis. Unfortunately, without early detection and treatment, this invasive cancer can insert itself deeply into a variety of tissues, including fat, muscle, and even bone.

A simple Google search for ‘melanoma’ (>34,300,000 results) demonstrates the importance of this cancer to human populations, as does the related search for ‘life expectancy’ (>34,400,000 results). One of the main goals of this review is to help increase the life expectancy of patients with skin cancer, resulting in causing the number of results for Google searches for ‘melanoma’ to plummet, as happened for ‘smallpox’ (>8,340,000 results), which was declared eradicated in 1980 [12].

## 2. Skin: Where One-Third of New Cancers Are Born

Skin, the human body’s largest organ, is complex. An adult has between 1.5 and 2 square meters of skin, responsible for about 15–17% of total body mass. In the skin, there are several distinct types of tissue organized by cell type into four primary layers. The stratum corneum, which forms a barrier against the environment, consists of dead keratinized cells. The subcutaneous tissue (subcutis) contains fibroblasts, adipose cells, and macrophages. The epidermis and dermis are both made up of epithelial, mesenchymal, glandular, and neurovascular components. As the outermost layer of skin, the epidermis, of ectodermal origin, is the body’s first point of contact with bacteria, viruses, chemicals, radiation, and humidity. The particular characteristics of the epidermis, both biological and physical, determine how it responds to and resists stressful environmental factors such as infectious pathogens, harmful chemicals, and UV light [13,14,15,16,17]. Constantly exposed to UV radiation from sunlight and other sources, the epidermis is highly susceptible to DNA damage. UVB radiation (280–315 nm), which makes up less than 2% of the UV rays in sunlight, is considered the major environmental cause of skin cancer. UVB is involved in both tumor initiation and promotion [18]. UVB completely penetrates the epidermis (0.03–0.13 mm thick) and is able to penetrate slightly below it into the dermis (1.1 mm thick). When this happens, the caused damage allows this area to become the focal point of a new cancer.

Malignant melanoma is the deadliest form of skin cancer. Over the last few decades, the incidence of this cancer, which is prone to metastasis and often difficult to treat, has increased steadily and significantly [19], so much so that globally, melanoma cases are increasing faster than those of any other cancer. Unlike cells that give rise to squamous cell and basal cell carcinomas, it is unknown where melanoma cells originate. There are several theories: dedifferentiated melanocytes may give rise to the precursors of melanoma, or these cells may develop from melanocyte progenitors in the bulge region of hair follicles or from Schwann cell precursors that derive from the neural crest [20,21,22,23]. On the other hand, squamous cell carcinoma is known to originate in the basal cell layer of the epidermis from progenitor/stem cells. While both squamous cell carcinoma and melanoma frequently metastasize, basal cell carcinoma does not [21,22]. The origins of basal cell carcinoma (the most common malignant skin cancer worldwide) are harder to pin down. It has been proposed that they develop either from cells in the bulge region of the hair follicle (an area rich in keratinocyte stem cells) or, like squamous cell carcinoma, in the basal cell layer of the epidermis from progenitor/stem cells [24]. Controversy surrounds the exact cellular origin of basal cell carcinoma. Evidence from studies points strongly at an origin in the stem cells within hair follicles and mechanosensory niches [25].

**Figure 2:**
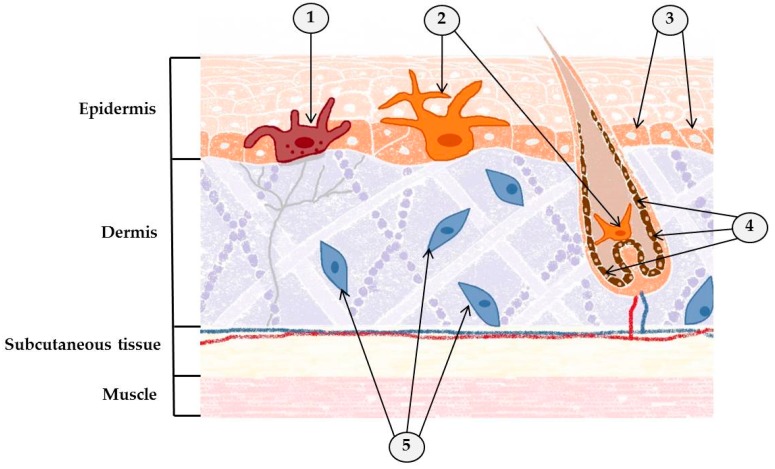
The origins of various types of skin cancer. 1—Merkel cell (Merkel cell carcinoma); 2—melanocytes (melanoma); 3—basal cells (basal cell carcinoma and squamous cell carcinoma); 4—keratinocytes (basal cell carcinoma); 5—fibroblasts (dermatofibrosarcoma protuberans).

Determining the identity of cancer cells in solid tumors has always been a challenge and remains so even now [26]. Fortunately, this is not the case with skin cancer. Indeed, as an organ, the skin lends itself to experimental investigation; it is much more accessible than the internal organs and is constructed of well-defined cellular compartments. In addition, genetically engineered mouse models that mimic the progression of melanocytic and epithelial skin cancers in humans have now been developed. This is important because we need a starting point from which to begin our assault on these aggressive cancers. For example, dermatofibrosarcoma protuberans, one of the unusual skin cancers mentioned previously, is known to be a mesenchymal tumor that originates in fibroblasts [27]. It is the most common of all the dermal sarcomas [28], usually affecting the dermis and subcutaneous tissue. Even though it is unusual, knowing the tissues that give rise to it and nourish it provide us with vital information about how to defeat it. Since the precise origins of some cancers remain elusive, there is still room for new and sometimes unexpected discoveries [29]. The origin of another unusual cancer, Merkel cell carcinoma, has been strongly linked to the Merkel cell polyomavirus. In a large number of cases (>80%), this cancer has been found to occur concomitantly with Merkel cell polyomavirus, a member of the normal human viral flora [30]. While it is most commonly believed that Merkel cell carcinoma derives from cutaneous Merkel cells or from some common precursor, its true origin is still a matter of debate [31,32,33] (Figure 2).

## 3. Ultraviolet Light Illuminates the Path of Skin Cancer

We tend to think of sunlight as visible light from the sun; white light made up of the different wavelengths of light that, with a prism, we can separate into a rainbow. However, solar radiation is the combination of this visible light and ultraviolet radiation (UVR), which comprises different wavelengths classified into three groups. The shortest and most dangerous wavelengths, <295 nm, are classified as UVC. Fortunately, the majority of UVC radiation is absorbed by the atmosphere before it reaches the Earth’s surface and our skin. Solar UVR, the radiation that does penetrate the atmosphere and reach the surface, has wavelengths >295 nm and is classified as UVA and UVB. While UVB (280–315 nm) represents only 5% of solar radiation, it is much more effective than UVA at damaging DNA. It causes cancer and sunburn in both humans and other animals, even though it only penetrates the upper layers of the skin. UVA radiation (315–400 nm), which represents 95% of solar radiation, penetrates deeper into the tissues. For the most part, it was believed that UVA caused little harm other than wrinkles and the cosmetically distressing signs of ageing of the skin. However, in epidemiological studies, it is difficult to separate the effects caused by UVB, UVA, and visible light. Therefore, these wavelengths are typically grouped together and treated as a unit [34]. Generally, UVR is an important risk factor for all skin cancers and is considered as a ‘complete carcinogen’ because it causes both general (nonspecific) skin damage and mutations and functions both as an initiator and as a promoter of tumors [35]. UVR is responsible for damage to the DNA (where it causes cyclobutane pyrimidine dimers to form) and gene mutations, including mutations to the p53 tumor suppressor genes involved in DNA repair and/or in the apoptosis of cells disabled by extensive DNA damage. By inducing the immunosuppressive cytokines interleukin-1 and tumor necrosis factor-alpha (TNF-α), UVR increases the levels of oxidative stress and related inflammatory responses. All of these negative effects play an important role in the photoaging of the skin and greatly increase the likelihood of initiation of skin cancer [36,37,38].

The amount of UVR that actually reaches the Earth’s surface is affected by many factors, including ozone depletion, UV light elevation, latitude, altitude, and weather conditions [39]. South America receives the greatest amount of ultraviolet radiation, particularly in the Peruvian Andes and throughout the west–central Altiplano region [40]. The following countries/cities have the highest UV levels: Australia (Darwin), Brazil (Rio de Janeiro), Cuba (Havana), Kenya (Nairobi), Madagascar (Tananarive), Mozambique (Maputo), Panama (Panama), Singapore (Singapore), Sri Lanka (Colombo), Thailand (Bangkok), and Vietnam (Hanoi) [41]. In 1992, Canada introduced the use of the UV Index (which ranges from 0 to 11+) as a way to address growing concerns about ozone depletion and how this might increase ultraviolet (UV) radiation. For example, between March and September, the UV Index in Vancouver (Canada) can be 3 or higher. When the UV Index is 3 or higher, the skin should be protected as much as possible [41]. It has been estimated that UV exposure, and thus a high UV Index, is one of the primary causes of 65% of melanomas and 90% of non-melanoma skin cancers [42]. Artificial UV rays are as dangerous as their natural counterparts; cosmetic tanning using indoor UVR predisposes its practitioners to skin cancer [39]. The soaring popularity of outdoor activities, in addition to indoor tanning devices, as well as the rapid depletion of the ozone layer, have all contributed to increased UVR exposure [43]. At the same time, it is important to remember there are significant positive effects that moderate doses of ultraviolet light contribute to all living creatures on the Earth, including humans. One of the best known and most positive effects of human exposure to both solar and artificial radiation is UVB-induced production of vitamin D in the skin. Vitamin D deficiency (≤20 ng/mL) is associated with increased incidence and worse prognosis of various types of cancer, including melanoma [44]. Successful treatments for several human skin diseases, such as psoriasis, vitiligo, atopic dermatitis, and localized scleroderma, include solar radiation (heliotherapy) or artificial UV radiation (phototherapy). UV also generates nitric oxide (NO), which research has shown may reduce blood pressure and generally improve cardiovascular health [45].

Different patterns of UV exposure, as well as different intracellular molecular pathways, affect the development of melanomas versus non-melanoma cancers. Squamous cell carcinoma can occur in the absence of UVR exposure; its occurrence has also been associated with scarring, nonhealing wounds, or chronic lesions caused by active or previous chronic immuno-inflammatory processes. This is not the case with basal cell carcinoma or melanoma [46]. However, when it comes to certain humans, these cancers are in agreement: squamous cell carcinoma, basal cell carcinoma, and melanoma all more frequently affect those who are elderly, red-haired, blue-eyed, and fair-complexioned [47]. Over the last few hundred years, European colonization and the emigration caused by wars and political unrest have led to humans with these vulnerable phenotypes moving to the areas with some of the highest rates of UV radiation exposure. Studies of the occurrence of melanoma across the South American continent have shown a strong correlation between white-skinned European ancestry and the risk of melanoma [48]. An increased risk of these malignancies has consistently been linked with variants of the melanocortin 1 receptor (MC1R) gene [49]. The MC1R, a melanocytic G protein-coupled receptor, has been shown to regulate skin pigmentation and the response to UV radiation, two factors heavily involved with the risk of melanoma. It is a highly polymorphic gene, frequently found in fair, UV-sensitive individuals (such as those described above) who are prone to melanoma as the result of defective epidermal melanization and the suboptimal DNA repair that occurs when the gene becomes dysfunctional. This is important because MC1R signaling, achieved through adenylyl cyclase activation and generation of the second messenger cAMP, is hormonally controlled by the positive agonist melanocortin [50]. This protects the skin against UV damage in two different ways. First, MC1R signaling increases the production and accumulation of eumelanin in the epidermis by inducing increased synthesis of melanocytic pigments. The resulting epidermal melanization blocks skin penetration by UV rays. When UV radiation cannot enter, the risks of superficial skin damage, mutagenesis, and the development of cancer are lessened. Second, MC1R signaling makes melanocytes more resistant to UV radiation by ramping up nucleotide excision DNA repair and oxidative resistance. The melanocyte stimulating hormone (MSH)–MC1R signaling axis plays a key role in determining both the type and the amount of melanin produced by melanocytes. As such, it represents a critical UV protective mechanism innate to the skin [35,50].

**Figure 3:**
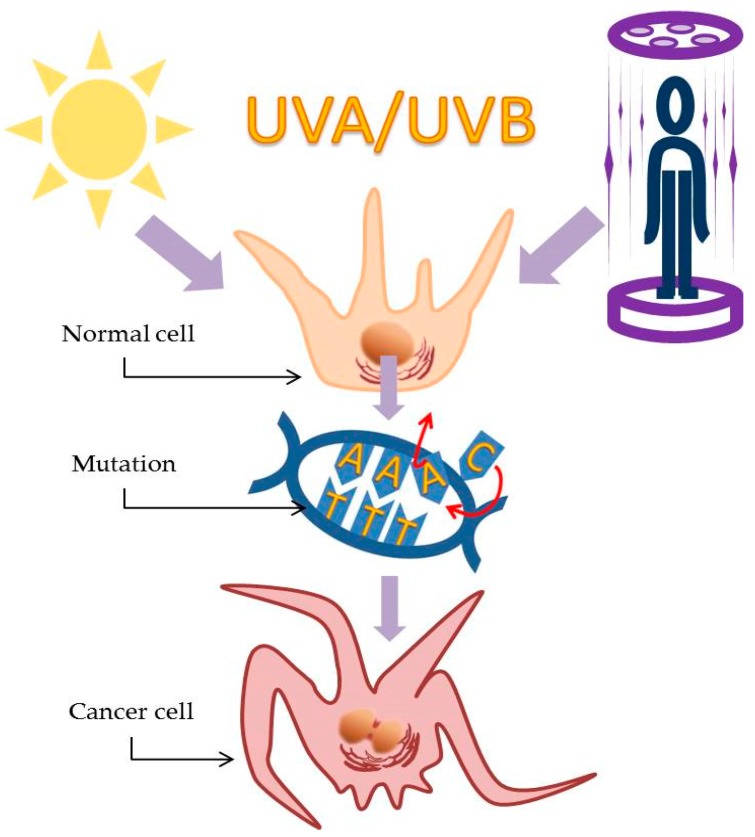
The effect of UV radiation on skin cells.

Exposure to UV radiation is one of the risk factors linked to the immunosuppression associated with Merkel cell carcinoma [51]. The mRNA transcript of Merkel cell polyomavirus (strongly linked to Merkel cell carcinoma) small t antigen had a dose-dependent increase after UV radiation (in the form of solar-simulated radiation) [52]. Moreover, Merkel cell carcinomas that are not associated with Merkel cell polyomavirus develop directly from UV-associated mutations [53]. There is a greatly increased incidence of Merkel cell carcinoma among fair-skinned individuals compared to its incidence in those with darker skin [54]. The lesions are most likely to develop on areas of the skin most frequently exposed to the sun, such as the head, scalp, neck, ears, and arms [55,56]. Unlike so many of the other skin cancers, the literature confirms that dermatofibrosarcoma protuberans is the only one without any obvious direct link to UV radiation. This unusual skin cancer tends to form lesions on the trunk (shoulders, chest, abdomen, back, buttocks), an area of the body not exposed to the sun with the same relentless frequency as the head and arms (Figure 3). It is also imperative to mention the important role of immunosupression in favouring the development of squamous cell carcinoma, which is extremely frequent in organ transplant recipients and represents a major cause of morbidity after organ transplantation [57]. Moreover, the role of certain viruses in promoting non-melanoma skin cancer is shown. The basis of such theory is represented by the genodermatosis epidermodisplasia verruciformis (EV), in which pathogenetic factors such as infection by beta human papillomaviruses as well as sun exposure are considered responsible for the malignant transformation of EV lesions to skin cancer within decades [58].

## 4. Help the Skin by Cutting the Cancer Out in Time

Clearly, the simplest and most effective way to get rid of skin cancer is to remove the tumor. The primary purpose of surgery in treating any cancer is to completely excise the tumor, thereby preventing local recurrence. Removing cancerous tissue is complicated by the very nature of skin: it covers, and interacts with, everything else in the body. A wide excision is used to treat melanomas by removing local micrometastases along with normal tissue that, while otherwise phenotypical, may hide genotypically abnormal cells in the surrounding cutis or the superficial lymphatics. It is difficult to find the balance between removing any and all cancerous cells and not causing unacceptable functional and cosmetic harm to the patient [59]. In addition, with respect to skin cancer, time is critical. If caught early, many melanomas can be managed by surgical excision alone. However, melanomas are highly invasive, metastasize quickly, and the prognosis for long-term survival is poor when the disease is advanced. Melanoma is one of the most aggressive malignancies with the lowest survival rates [60]. Progress has been made with the use of targeted therapies [61,62,63,64,65] and immunotherapies [66,67], but melanoma remains notoriously difficult to treat once it has spread beyond its original site. A large number of clinical trials has been carried out testing all kinds of anticancer strategies. These strategies range from different types of surgery to immuno-, radio-, and chemotherapy [68,69,70,71], and yet the average rate of survival is still only 6 to 10 months. The prognosis for melanoma would be less devastating if the malignant melanocytes were detected at a much earlier stage, before becoming invasive. However, melanomas have several characteristics that make early detection difficult.

**Figure 4:**
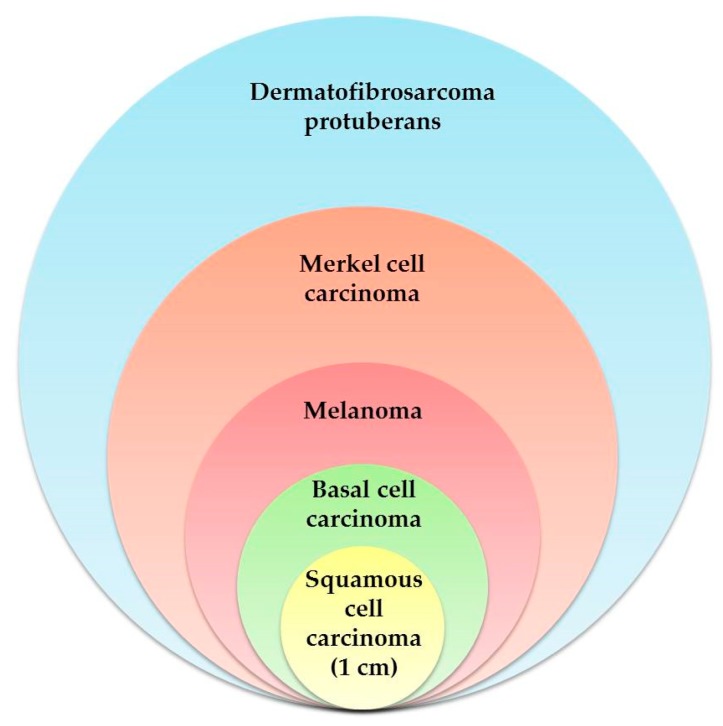
The relative size of peripheral margins in the removal of various types of skin cancer.

In the early stages of melanoma, surgery often completely removes the cancer. Patients with Stage I/II melanomas have experienced successful eradication of their cancers following complete surgical excision of their primary tumors. In patients with lymph node infiltrations (Stage III melanoma), this surgery significantly extends their long-term survival [72]. However, once the cancer has developed metastases, surgery alone is no longer the cure. Metastatic melanoma continues to have a poor prognosis, unlike localized melanoma, which responds well to surgery, because melanomas have a tendency to develop resistance to chemotherapy drugs, often after only a few cycles of treatment [73]. In addition to being aggressive and drug-resistant, melanoma lesions can remain unnoticeable or asymptomatic for extended periods of time, which presents even well-trained dermopathologists with a challenge [74,75]. By the time the cancer is discovered and diagnosed, it can have spread in ways that complicate the treatment plan. In one retrospective study of a series of 576 patients from a single center, the width of the excision mentioned earlier was varied. All of the patients had 1–2-mm-thick melanomas; while those with 1-cm margins were observed to have a small increase in local recurrence compared with patients with 2-cm margins, the width of the excision had no impact on overall survival [76]. Common sense would seem to dictate that excision with wider margins more completely removes microscopic disease than that with narrower margins, which might allow some cancer cells to remain. The long-term results from trials carried out to specifically test this idea failed to demonstrate that a wider excision independently predicted melanoma-specific survival [77]. One could conclude, and many have, that there is little evidence to support the accepted paradigm and believe that any width of histologically clear excision margin is acceptable [78] (Figure 4).

Non-melanoma skin cancers occur much more frequently than melanomas; fortunately, they respond much better to treatment and have a better long-term prognosis. Local control measures, such as resection, Mohs micrographic surgery, or cryosurgery, are often completely successful treatments for these cancers [79]. In addition, nonsurgical procedures such as systemic and topical pharmacotherapy, cryotherapy (CT), photodynamic therapy (PDT), laser therapy, and radiotherapy (RT) have also been used successfully [80,81,82,83,84]. When diagnosed early, both basal cell carcinoma and squamous cell carcinomas are easy to treat and rarely fatal. However, in a small number of cases, both of these cancers have demonstrated the potential to metastasize and have been the cause of death. Moreover, both the developmental stages of these cancers and their subsequent treatments can be painful and cause disfigurement [85]. Early recognition is the key to preventing development of the advanced stages of the disease. Many surgical and oncological organizations have established guidelines for the treatment of non-melanoma skin cancers; unfortunately, these guidelines vary greatly and are not always consistent. Generally, the margin required for high-risk basal cell carcinoma lesions is usually not more than 1.5 cm, and for high-risk lesions of squamous cell carcinoma, is usually not more than 1 cm [86].

The standard of surgical care for Merkel cell carcinoma is still focused on local/regional intervention [87]. Excision margins as wide as 3 cm have been reported in the literature (range: 1 to 3 cm) in attempts to successfully reduce local recurrence [86]. Almost all patients who experience recurrence do so within the first 2 years of the initial treatment. In one study, most recurrences (64% of first-time recurrences) were attributed to metastases to the lymph nodes. The majority of patients (76%) in that study had Stage I carcinoma, with an overall survival rate of 81%. Unfortunately, for the rest of the patients, whose cancers had begun to spread, the progression of the disease led rapidly to death. Their median survival rate following the development of metastases was 5 months [88]. Since the metastatic cells drain into the lymph nodes, pathologic evaluation of the nodes increases the accuracy of prognoses in patients with Merkel cell carcinoma [89].

Some cancers are known to require drastic excision of several types of tissue in addition to skin. We mentioned dermatofibrosarcoma protuberans earlier; locally aggressive, this sarcoma grows slowly and is known to have highly irregular tumor shapes with eccentric projections and a high rate of recurrence [90,91]. Although this cancer is rare and behaves differently than some of the other skin cancers, its aggressive nature means that correctly determining the treatment margins is crucial. Dermatofibrosarcoma protuberans definitely demands a three-dimensional approach to surgical excision; the established guidelines recommend peripheral margins no less than 1 cm and up to 4-cm wide and deep margins that dive well down into the fascia [86]. Although this cancer occurs primarily on the trunk, recurrences tend to occur on the head, neck, and other extremities, probably because it is difficult to achieve wide enough margins in these areas [92,93]. Fortunately, the majority of cases of recurrent dematofibrosarcoma protuberans can be cured with re-excision [94]. The Mohs–Tübingen technique is a variant indicated for very large excisions that allows a complete eradication of the tumour, preserving healthy tissues [95].

## 5. What Happens if the Surgeon Does Not Succeed?

“Cut as soon as possible and as many times as needed”: this would seem to be the modern mantra for the treatment of skin cancer. When excision works and causes minimal trauma and scarring, the cancer survivors are grateful and all is well. Unfortunately, it does not always work. For this reason, among others, we asked: are there any alternatives or additional medical practices that could be used in conjunction with (or even substitute for) surgery that could decrease the probability of recurrence, or even prevent lesions from developing in the first place? Notable results have been achieved with the use of chemoprevention and chemotherapy, but the possibility of preventing/curing serious skin cancers such as melanoma and its ‘younger sister’, Merkel cell carcinoma, remains elusive. These two skin cancers with the highest death rates will be the focus of our investigation in this section.

### 5.1. Botanicals as a Hope

Despite the development of and research using a plethora of chemopreventive agents (e.g., statins, curcumins, resveratrol, silymarin, epigallocatechin-3-gallate, selenium-containing agents, nonsteroidal anti-inflammatory drugs, beta carotene, celecoxib, alpha-difluoromethylornithine, sunscreens, betulinic acid, and vitamin D), none of them, singly or in concert, have been effective at preventing melanoma. Chemopreventive interventions can be introduced at different stages of carcinogenesis. Primary chemoprevention in normal tissue strives to inhibit the formation of mutagenic molecular species and/or facilitate the repair of any damage they have caused. The aim of secondary chemoprevention is to disrupt the progression of the disease by slowing, blocking, or reversing the conversion of premalignant cells to melanoma. Tertiary chemoprevention encompasses treatments used to prevent the recurrence of melanoma in patients previously treated for the disease. Time and time again, nature has provided compounds that prevent, diminish, or obliterate cancer. Thus, the search continues for different, more potent natural compounds or combinations of compounds with greater chemopreventive efficacy [96,97,98]. Examining naturally occurring compounds is highly sensible. Several decades ago, 75% (90 of 121) of the prescription drugs being used to treat cancer treatment were derived from plants [99]. Three-quarters of these were discovered when researchers began to explore the claims of medicinal folklore and the history of tribal, plant-based medicines [100]. Trying to pinpoint which component in a naturally occurring compound is the one responsible for the useful effect is tricky. Often, even when results are promising, we do not know how or why. Studies of selenium-containing isoselenocyanates and isoselenoureas in laboratory-generated skin reconstructs, as well as in xenografted animal models, have produced exciting data. While this is encouraging, the safety and efficacy of these agents for use by humans require further studies. There are other naturally occurring compounds, such as curcumins and epigallocatechin-3-gallates, that also deserve further study with respect to melanoma prevention. We need to find compounds that are cost-effective to produce, effective when used, and able to eradicate the cancer without seriously disabling the patient [96].

### 5.2. Mutations as a Grounding for Efficient Treatment

A combination of surgery, radiotherapy, and/or chemotherapy comprises the current treatment strategies for both advanced melanoma and for Merkel cell carcinoma. Unfortunately, melanoma consistently responds poorly to chemotherapy while still managing to subject patients to severe adverse side effects. Response to treatment with dacarbazine, the most commonly used therapy for metastatic melanoma, ranges from ~10 to 20% [101,102]. Several studies have yielded even lower response rates for treatment with dacarbazine; complete responses are rare [103,104]. For patients with the B-RAFV600E mutation, therapies specifically targeting the MAPK (mitogen-activated protein kinase) pathway, such as vemurafenib (PLX-4032), dabrafenib, and trametinib, have significantly improved their overall survival [105]. B-RAF is a serine/threonine protein kinase that activates the MAP kinase/ERK (extracellular signal-regulated kinase) signaling pathway. Inside cells, the B-RAF protein is involved in the signaling that directs cell growth. Approximately 50% of melanomas have been found to harbor activating B-RAF mutations. Over 95% of B-RAF mutations include the V600E mutation, which cause the substitution of glutamic acid for valine at residue 600 of the protein [106]. As is the case with so many skin cancers, exposure to UV radiation plays a role in the genesis of B-RAF mutations in cutaneous melanoma [107]. For example, B-RAF V600E mutation has been implicated in different mechanisms underlying the initiation and development of melanoma, owing mostly to deregulated activation of the downstream MEK/ERK effectors [108]. While these drugs contribute to improved survival, the swift initial response to treatment with vemurafenib (PLX-4032), dabrafenib, or trametinib cannot be sustained because cancers rapidly become resistant to them [109]. Activation of STAT3 (a signal transducer and activator of transcription) and its signaling pathways have been implicated in melanomas that become resistant to vemurafenib [110]. For this reason, it is important to note that suppression of STAT3 activity disrupts B-RAF V600E-mediated induction of antiapoptotic proteins, seriously affecting the survival of melanoma cells [111]. It is no surprise, therefore, that these treatments have no effect in patients lacking the B-RAF V600E mutation [109].

It is noteworthy that the prognosis for metastatic melanoma patients with B-RAF mutations improves dramatically when immunocheckpoint inhibitors are used. When skin cells are damaged, for example by UV radiation, activated T-cell lymphocytes replicate and migrate to the damaged site. CD4+ helper T cells and CD8+ cytotoxic T cells (CTL) provide one of the main defenses against cancer cells: they detect and kill cancer cells in their role as managers of humoral and cell-mediated responses [112]. Some regulatory mechanisms exist to control the intensity and duration of the T-cell response or to help maintain cellular equilibrium. For instance, lymphocytes can monitor their environment and overexpress coinhibitory immune checkpoint molecules, such as cytotoxic T-lymphocyte-associated protein 4 (CTLA4), to antagonize the costimulatory signals activating them. Cancer cells overexpress two programmed death ligands (PDL1 and PDL2); lymphocytes express a receptor, programmed cell death protein 1 (PD1), that readily recognizes its ligand. This can lead to exhaustion as the lymphocytes respond to the cancer cells. The FDA has approved only one anti-CTLA-4 drug, ipilimumab. Unfortunately, despite whether it is used as a first-line or second-line therapy, the patient survival rate 3 years after treatment is only 20% [113]. The clinical application of these drugs needs further elucidation; much energy is being spent researching how to increase the efficacy of immunotherapy.

Another cohort of mutations driving the development of melanoma is found in the neuroblastoma RAS viral (v-ras) oncogene homolog (NRAS), which codes for a small guanine triphosphate (GTP)-binding protein. RAS oncogenes with activating mutations have been observed in a third of all human cancers; 15–20% of melanomas carry NRAS mutations, most frequently at hotspots in exon 2 (codon 61) (NRAS Q61L/R). Compared to melanomas without any NRAS mutations, the subset of melanomas with NRAS mutations is more aggressive and inevitably associated with poorer outcomes [114]. NRAS mutation status has been shown to be a useful independent predictor of shorter survival rates following diagnosis with Stage IV melanoma [115]. Although increased use of immune checkpoint inhibitors and targeted therapies for B-RAF-mutant melanomas has transformed the treatment of certain metastatic melanomas, the ideal treatment for NRAS-mutant melanomas remains unknown [114]. Since patients with mutant NRAS tumors tend to be older, with a history of chronic ultraviolet (UV) exposure, their cancers are more challenging to treat successfully [115,116,117]. Clinical trials are being carried out to investigate a large number of the newer targeted therapeutic strategies, specifically mono- and combination therapy with MEK inhibitors, which appear promising. While immune-based therapies are not genotype-specific, compared with their efficacy in other melanomas, they seem to be at least as effective, possibly more so, in the subset with NRAS mutations [114].

**Figure 5:**
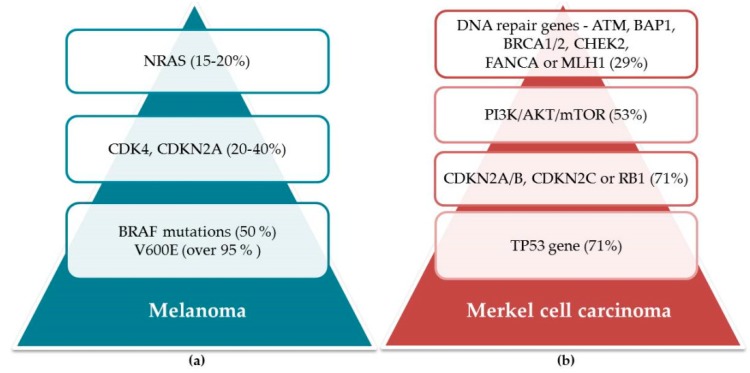
Mutations resulting in melanoma (**a**) and Merkel cell carcinoma (**b**).

Few advances have been made in the chemoprevention of Merkel cell carcinoma, which is also the case with melanoma. The pathogenesis of Merkel cell carcinoma is not well understood; despite the upswing in mutational analyses, a set of signature mutations has yet to be identified in the majority of cases. While some mutations, including TP53, retinoblastoma, and PIK3CA, have been documented in certain subsets of patients, it is likely that other mechanisms are also involved, including some patients infected with the Merkel cell polyomavirus, dysregulated immune surveillance, epigenetic alterations, aberrant protein expression, post-translational modifications, and microRNAs [118]. In addition, several other molecular abnormalities have been reported in Merkel cell carcinomas, including overexpression of Hedgehog (Hh) signal pathway proteins, telomerase activation (TERT), chromosomal abnormalities [119], and microRNA alterations [120]. Those with compromised immune systems are at greater risk; the immunosuppression accompanying HIV infection increases the relative risk of Merkel cell carcinoma ~13-fold, and that experienced by solid-organ transplant patients by 10-fold [121,122]. As if one cancer were not enough, patients with chronic lymphocytic leukemia (CLL) are also at increased risk for Merkel cell carcinoma [123]. It is not well understood how immunosuppression and Merkel cell carcinoma are related. However, the fact that in ~80% of Merkel cell carcinomas, those with the disease were found to have first been infected with the Merkel cell polyomavirus (MCPyV) provides evidence for a potential mechanism effecting the malignant transformation [124]. The mechanisms responsible for MCPyV-negative Merkel cell carcinoma oncogenesis, which are also poorly understood, may involve somatic mutations in tumor suppressors, such as RB1 and TP53, or epigenetic alterations that cause aberrant expression and activity in oncogenes [125,126]. One study identified 30 genes with aberrations and 60 distinct molecular alterations among the patient population. The most common abnormalities, found in 71% of the patients, involved the TP53 gene and the cell cycle pathway (CDKN2A/B, CDKN2C, or RB1). Abnormalities also were observed in the PI3K/AKT/mTOR pathway (53%) and DNA repair genes (ATM, BAP1, BRCA1/2, CHEK2, FANCA, or MLH1) (29%) (Figure 5). Possible cognate targeted therapies, including FDA-approved drugs, could be identified in most of the patients. The authors concluded that Merkel cell carcinomas were characterized by multiple distinct aberrations that were unique in the majority of analyzed cases. Most of the patients had alterations that were theoretically treatable. These results provide a framework for investigating tailored combinations of matched therapies in Merkel cell carcinoma patients [127].

The response of Merkel cell carcinoma to treatment modalities is variable, along with its prognosis, which means that clinical and histologic characteristics are of limited use when it comes to predicting outcome. Underlying the perplexing natural history of Merkel cell carcinoma are several poorly understood factors. These include unique differences in chromosomal abnormalities, genetic mutations, expression profiles, and epigenetic controls of individual tumors. When a better understanding of Merkel cell carcinoma can be achieved at the molecular level, it will increase our chances of predicting how these tumors will respond to aggressive surgery, the prognosis for patients, and how to develop more effective targeted therapies [118]. An important factor in the analysis of mutations in Merkel cell carcinoma is MCPyV status. Discovered in 2008, the Merkel polyomavirus is one of 13 known polyomaviruses that naturally infect humans. Among these, however, it is the only human polyomavirus believed to be involved in tumorigenesis [128]. Why is the infection rate with this virus higher in Merkel cell carcinoma patients? What sparks oncogenic transformation in infected patients? The answers to these questions are unknown, although it is likely that immunocompromise plays a role [129]. In immunocompromised patients, the incidence of Merkel cell carcinoma is greater by 15-fold, increasing to 30-fold in patients with other blood cancers [123,130]. Chemotherapy is one of the most common treatments used for patients with metastases. Current systemic chemotherapies include platinum, administered with or without etoposide, as well as cyclophosphamide, doxorubicin, and vincristine [131,132,133]. The responses achieved with these cytotoxic agents are modest, at best; the median progression-free survival is 3 months. Unfortunately, many cancers develop resistance to the drugs following two to three treatment cycles. For this reason, treatments that rely on different procedures and/or on agents other than drugs have been investigated, including radiation therapy [133,134]. Immunotherapy using a humanized anti-PD1 antibody (for example, systemic pembrolizumab (MK-3475)) [135] and targeted molecular therapy are two other investigational approaches that have been used to treat metastatic Merkel cell carcinoma [136,137,138,139]. The disease is rare; therapeutic response data often derive from case reports and studies of retrospective series rather than clinical trials, making it challenging to define the role of chemotherapy in the management of advanced Merkel cell carcinoma. In fact, there are no drugs approved by the FDA that specifically target Merkel cell carcinoma [127].

## 6. Keep Silencing: Approaches for the Treatment of Melanoma and Merkel Cell Carcinoma Using Antisense Oligonucleotides

For both metastatic melanoma and Merkel cell carcinoma, there is definitely room for improvement with respect to treatment. The need to develop novel agents for the treatment of these cancers is urgent. Changes in the genetic code of certain genes in the human dermis and epidermis are the source that ‘gives birth’ to skin cancer. It seems obvious that it makes sense to apply techniques used to manage other types of ‘poorly working genes’ by enlisting the help of the universal molecules that control all cells: nucleic acids, particularly antisense oligonucleotides. Current use of antisense oligonucleotides in therapeutic medicine may seem exotic, as they were originally developed for and are being used to treat quite rare diseases, such as familial hypercholesterolemia [140], spinal muscular atrophy [141], and Duchenne muscular dystrophy [142]. As we examine how they might help address two difficult cancers, melanoma and Merkel cell carcinoma, it is worth mentioning these drugs and their developers, because they too “chose to address difficult conditions, with seemingly unreachable goals, where conventional approaches had not noticeably succeeded” [143].

Antisense oligonucleotides (ASOs), short single-stranded polymers based on DNA or RNA chemistry and synthesized in vitro, silence gene expression by binding to an RNA target in a sequence-specific manner. The combination of nitrogenous bases in a target sequence significantly affect the functional activity and selectivity of the action of ASOs. Clinical experiences with the first generation of antitumor ASOs proved to be disappointing [144]. As a result, scientists have included many chemical modifications (phosphorothioates, 2’-O-methyl oligonucleotides, locked nucleic acids, morpholino oligomers, etc.) in the ensuing generations of ASOs to improve binding properties and the ability of ASOs to penetrate cells, to increase their stability in vivo and their efficacy, and to diminish unintended nontarget effects [143]. Fomivirsen, the first antisense drug made with a phosphorothioate backbone, was approved in 1998 by the U.S. Food and Drug Administration (U.S. FDA) and in 1999 by the European Agency for the Evaluation of Medicinal Products (EMEA) for the treatment of retinitis cytomegalovirus (CMV) in patients with AIDS. Although fomivirsen was administered locally as an intravitreal injection, its use demonstrated the possibility that drugs developed from antisense oligonucleotides could be administered systemically in the treatment of human diseases [145]. Several drugs (Kynamro^®^, Spinraza^®^, EXONDYS 51^TM^, etc.), generally administered via intravenous infusion, are approved today by the FDA [143]. Unfortunately, oligonucleotide-based therapeutics has not yet delivered a clinical drug to the market in the cancer field [146].

**Figure 6:**
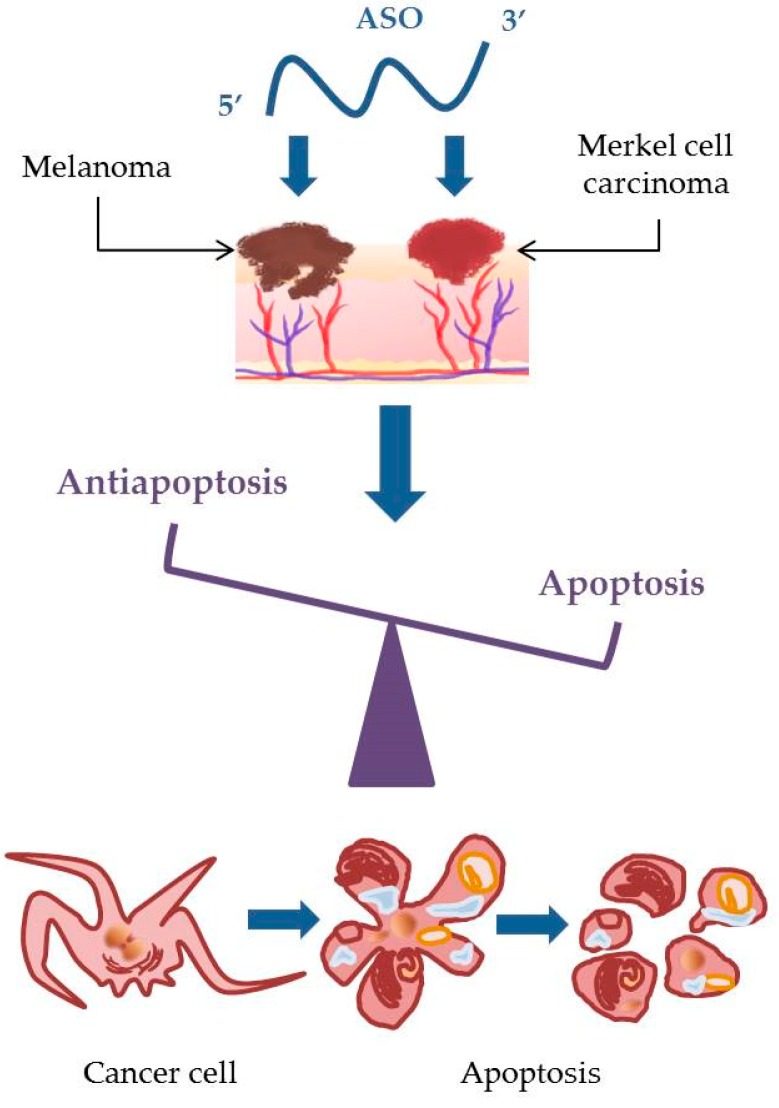
The effect of antisense oligonucleotides on skin cancer cells. ASO: antisense oligonucleotide.

Antisense describes a pharmacologic strategy that blocks the translation of specific proteins from mRNA, thus decreasing their expression (Figure 6). A specific and pertinent example is the malignant transformation of normal cells, which requires the inactivation of apoptosis. Healthy normal cells use apoptosis to control cell damage. When they lose this important function, they can become cancerous. Many chemotherapeutic agents induce apoptosis [147]; when melanoma cells are unable to undergo apoptosis in response to this cytotoxic assault, the treatment fails [148]. Data from genetic, functional, and biochemical studies suggest that melanomas have learned how to use this reduced or complete inability to undergo apoptosis as a way to circumvent the actions of chemotherapy drugs and that they have developed strategies to protect the pathways responsible for survival and proliferation, allowing the melanoma to thrive [149].

The development of drug resistance common to melanomas has been attributed in part to overexpression of BCL-2, an antiapoptotic protein integral to the overall process of apoptosis regulation [73,149]. BCL-2 is located deep in the mitochondrial membrane, where it blocks the release of cytochrome C [150], canceling out the attempts of cytotoxic chemotherapy to trigger cancer cell death by activating an apoptotic cascade initiated by mitochondrial release of cytochrome C and activation of caspase 9 [151]. Antiapoptotic BCL-2 family members are divided into two subclasses, one comprising BCL-2, BCL-X_L_, and BCL-W, and the other MCL-1 and BCL-2A1. Both subclasses must be neutralized before apoptosis can be induced [152]. Clinical studies investigating the effects of BCL-2 antisense therapy in patients with melanoma have shown promise. The data suggest that BCL-2 and BCL-XL are promising targets for the development of antisense therapies for melanoma, including additional clinical benefits from the simultaneous downregulation of their expression [148]. One example of this is oblimersen sodium (Genasense; Genta International Inc, Berkeley Heights, NJ), an 18-base phosphorothioate antisense oligonucleotide that binds to the first six codons of the BCL-2 mRNA open reading frame and mediates the cleavage of RNA by RNase H. Oblimersen downregulates the expression of the BCL-2 protein and increases chemotherapy-induced apoptosis in human cancer xenografts [153,154]. Oblimersen is rapidly metabolized into metabolites and shorter versions of the parent molecule. This is carried out by exonucleases that sequentially cleave single nucleotides from the parent oligonucleotide, which results in a mononucleotide metabolite and the parent molecule shortened by 1 nucleotide (e.g., N-1 (a 17-mer) and N-2 (a 16-mer)) (data on file, Genta Incorporated). In pharmacokinetic studies, analysis of the primary metabolites (N-1 and N-2) has shown them to be biologically active. In some published studies, the pharmacokinetic parameters reported for oblimersen comprised the sum of oblimersen and its metabolites because the analytical methods used were unable to resolve the parent molecule from the chain-shortened metabolites [155]. Data from one study show that the addition of oblimersen to dacarbazine in the treatment of advanced melanoma significantly improved multiple clinical outcomes and increased the overall survival of patients whose baseline serum LDH was not elevated [151]. Initial clinical studies carried out using a continuous 14-day subcutaneous infusion of oblimersen found dose-limiting toxicities of fever, thrombocytopenia, and fatigue [156].

Another antiapoptotic protein, survivin, may prove useful in developing therapies using antisense oligonucleotides in melanoma. Survivin is a member of the inhibitor of apoptosis protein (IAP) family. Expression of survivin is high in embryonic and fetal organs and in various human cancers, including melanoma, but is minimal or absent in adult normal cells and undetectable in most terminally differentiated human tissues [157,158,159]. Survivin binds to procaspase-9 in association with a cofactor and selectively suppresses the mitochondria/cytochrome C apoptosis pathway [160]. During mitosis, overexpressed survivin associates with microtubules of the mitotic spindles and demonstrates oncogenic properties by overriding the G2–M phase checkpoint [161]. LY2181308 (Eli Lilly and Co.) is an antisense oligonucleotide molecule designed to inhibit survivin. This 18-mer 2’-O-methoxyethyl (MOE)-modified second-generation ASO was specifically developed to inhibit expression of survivin in tumor cells. When LY2181308 binds to the translation initiation codon on survivin mRNA, it blocks translation, which leads to degradation of the transcript [162]. In a dose-escalation study of protein expression and apoptosis, patients received intravenous LY218308 before and after their breast tumors were biopsied. The investigators hoped to demonstrate reduced survivin protein expression and restored in vivo apoptotic signaling [163]. Use of these oligonucleotides includes some caveats; their stability is limited in vivo and they were inefficient when targeting and neutralizing survivin mRNA [164]. In addition, in patients receiving long-term treatment with antisense oligonucleotides, kidney function should be monitored frequently. This is particularly important when they are being treated with angiotensin-converting enzyme inhibitors or nonsteroidal anti-inflammatory drugs in combination with ASOs that target survivin [165].

Decreased apoptosis is a hallmark of Merkel cell carcinoma regardless of the patient’s Merkel cell polyomavirus status. High levels of expression of the BCL-2 protein in Merkel cell carcinoma may contribute to tumor growth by rescuing the transformed cells from apoptosis. For this reason, finding a way to modulate the expression of BCL-2 offers a promising strategy for treating Merkel cell carcinoma; the results from two independent studies found overexpression of antiapoptotic BCL-2 in 75% of the Merkel cell carcinoma tumors studied [166,167]. In one study, human Merkel cell carcinoma was grown in SCID (severe combined immunodeficient) mice (characterized by an absence of functional T cells and B cells) and BCL-2 antisense phosphorothioate oligonucleotides were used. Western blot analysis of the tissue from oligodeoxynucleotide-treated tumors showed a 30% reduction of BCL-2 levels of in the antisense group, a reduction not found in any other treatment group. While this reduction of BCL-2 levels seems rather small, it became obvious that the growth of tumors in the antisense group was also reduced after the second week of treatment [168]. However, when tested in a Phase II trial in humans, these same antisense oligonucleotides demonstrated very little, if any, efficacy in patients with Merkel cell carcinoma [169]. From the sequence specificity of the effects observed in Merkel cell carcinoma, it would appear that the BCL-2 antisense oligonucleotide used in the study employed an antisense mechanism of action; however, additional non-antisense interactions cannot be excluded [170].

## 7. Future Perspectives

Few attempts have yet been made to counteract skin cancer using antisense oligonucleotides. With difficult cancers such as melanoma and Merkel cell carcinoma, gene silencing remains an effective prospective strategy to limit disease progression. Inhibition of the expression of apoptosis/antiapoptosis system proteins, such as BCL-2 and survivin, could reverse or even prevent the disease process.

Cutaneous delivery of antisense oligonucleotides provides a simpler, less stressful alternative to intravenous injection with an excellent potential in the treatment of skin diseases. The skin, which is easily accessible, is the perfect target for gene-silencing strategies developed to treat localized skin diseases including skin cancer, psoriasis, and atopic dermatitis [171,172]. Compared with other organs, it is easier and by far more practical to assess the efficacy of treatment by visual inspection or biopsy of the skin [171]. In addition, compared with other routes of delivery, the skin has relatively fewer nucleases [173]. Topical application of antisense therapy to the skin allows it to be confined to the affected area, reducing the likelihood of either localized or systemic toxicity, such as the thrombocytopenia caused by phosphorothioate oligonucleotides [171]. In a clinical trial carried out in humans, administration of the phosphorothioate oligonucleotide ISIS 2302 caused dose-related elevations in activated partial thromboplastin time up to 10 s in length [174]. In studies carried out in animals, systemic administration of phosphorothioate oligonucleotides resulted in other, nonspecific toxicological problems. Intravenous administration is more likely than topical delivery to result in systemic absorption of oligonucleotides because of the relatively low lipophilicity of these molecules [171]. However, the high molecular weight (≥3300 Da) and negative charge commonly associated with an ASO limit its effective delivery through the skin. Delivery of an ASO is also limited by poor cellular uptake and entrapment in the harsh endolysosomal compartment of the cell [175]. Degradation of an ASO by nucleases occurs rapidly, which means that the systemic half-life ranges from a few minutes to hours [176]. Chemical modification of the phosphate backbone improves the stability of antisense oligonucleotides, but membrane permeability, which is vital, remains a challenge [177]. Development of efficient carriers to improve membrane permeability and cell uptake of ASOs is of paramount importance.

Antisense nucleotides cannot begin attacking cancer cells until they can reach them. When it comes to penetrating the skin, several obstacles need to be overcome. A variety of techniques that increase the ability of antisense oligonucleotides to penetrate the skin has been investigated [178]. Chemically, use of skin-penetrating peptides and chemical enhancers such as liposomes has been shown to enhance the skin penetration by ASOs and siRNA [179,180]. Active physical techniques that allow passage through the stratum corneum into the viable dermal tissues below include use of microneedles, electroporation, laser ablation, and low-frequency ultrasound [181,182,183,184]. In one study, it was demonstrated that dendrimers (an example of a chemical enhancer) are promising nanocarriers for penetrating the skin and delivering topical gene silencing nucleotides. An ASO–dendrimer complex delivered iontophoretically (an example of an active, physical method that navigates past the stratum corneum) to porcine skin successfully reached the viable epidermis. In contrast, passively delivered free or dendrimer-complexed ASO remained localized to the stratum corneum and therefore largely ineffective. The dendrimer complex significantly enhanced uptake of ASO by the cells, which allowed the complex to suppress the overexpression of BCL-2 within the cell. In skin cancer studies using a mouse model and a combination of chemical and active physical methods, an iontophoretically delivered ASO–dendrimer complex reduced tumor volume by 45%. It also reduced the protein levels of BCL-2 and caused significant apoptosis in the skin tumor cells [185]. In another study [186], the authors demonstrated that they could effectively deliver melanoma treatment in vivo using nanoparticles. The surface of single-walled carbon nanotubes was treated with a polymeric surfactant, polyethyleneimine (PEI), to make the inert surface ‘functional’, i.e., more attractive to small organic molecules. These functionalized carbon nanotubes were used in a mouse melanoma model to deliver anti-B-RAF siRNA, which resulted in the silencing of B-RAF and attenuated tumor growth.

Another prospective method of delivery of antisense oligonucleotides, which has not yet been tested on melanoma or Merkel cell carcinoma, is use of a specific cream or ointment formula as the vehicle. These creams and ointments cannot reach the living epidermis and dermis to exert their anti-inflammatory effects if the large anionic molecules within them cannot penetrate the tough layers of the stratum corneum [172]. The epidermis is made up of keratinocytes busy moving upward to eventually form the environmental barrier of the stratum corneum. Within the epidermis, these keratinocytes are densely packed together by cell–cell tight junctions, which use cell adhesion to obliterate intercellular space. Paracellular transport in the epidermis is inhibited by the channel-forming tight-junction proteins claudin-1, claudin-4, occludin, and zonula occludens-1 [187]. This makes both vertical and lateral transport difficult for a variety of molecules. To be effective, treatments comprising nucleic acids and other large macromolecules, along with nanoparticles, nucleic acid–lipid complexes, and other drug carriers, need to be able to move more deeply into the skin. They also need to be able to reach peripheral areas of the skin by moving laterally from the site of administration. The barriers created by the stratum corneum and the epidermal transport, which exist to protect the body from invasive molecules, in this case must be overcome if treatments such as nucleic acids are to be delivered to diseased areas [188].

Several groups have evaluated the accumulation of oligonucleotides in the skin following topical application. It is difficult to draw concrete conclusions from their results, which tend to vary depending on the chemical modifications incorporated into the oligonucleotide sequence and whether psoriatic or normal skin was used [173,189,190]. In one study carried out on human skin transplanted onto severely compromised immunodeficient mice, researchers applied a cream formulation containing a 20-nucleotide phosphorothioate intercellular adhesion molecule-1 antisense oligodeoxynucleotide. The cream contained glycerol monostearate (10%), hydroxypropyl methylcellulose (0.5%), isopropyl myristate (10%), hydroxypropyl methylcellulose (0.5%), isopropyl myristate (10%), methylparaben (0.5%), propylparaben (0.5%), polyoxyl-40-stearate (15%), and water. This antisense oligodeoxynucleotide effectively inhibited TNF-α-induced expression of intercellular adhesion molecule-1. The effects of this topical cream, which resulted from reduced levels of intercellular adhesion molecule-1 mRNA in the skin, were concentration-dependent and sequence-specific. While intravenous administration failed to show any pharmacologic effects, possibly because the concentration of the oligodeoxynucleotide in the skin was insufficient, topical administration resulted in a rapid and significantly higher accumulation of the drug in the epidermis and dermis. These results strongly indicate that antisense oligonucleotides can be delivered to target sites on the skin via topical application. This is valuable information that could revolutionize the treatment of psoriasis and other inflammatory skin disorders [173].

While our skin has evolved to present a serious impediment to foreign molecules seeking entrance to our bodies, one that is quite tough to breach, it does have holes: pores and hair follicles. In a study investigating ASOs carried in a lipophilic vehicle, it was observed that these topically applied oligonucleotides accumulated in the hair follicles, from which area they were trafficked into the dermis along with their vehicle. The authors also observed that while the ASO as formulated was unable to penetrate the skin of hairless mice, the results were dramatically different when the same ASO cream was applied to the hair-clipped skin of BALB/C mice. These experimental data led the investigators to hypothesize that following topical application of an ASO formulation, the hair follicle is a route of ASO trafficking into skin [172], which confirms the results of other researchers who have also demonstrated that large macromolecules could be delivered into the skin via hair follicles [191,192]. While ASOs have been tested in both cream and simple saline formulations, successful dermal distribution of ASOs has only been observed when they are applied via cream formulations [172].

**Figure 7:**
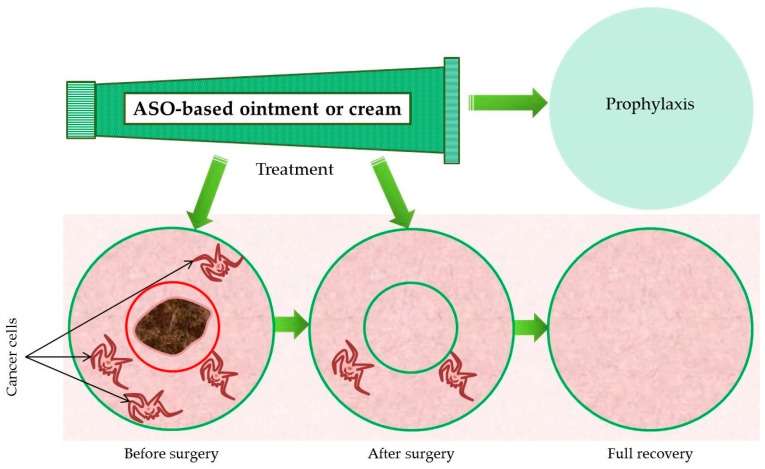
Methods of applying a cream or ointment based on antisense oligonucleotides in the treatment of melanoma.

Our research group has concentrated on the creation of an antimelanoma ointment with antisense BCL-2 and antisense survivin phosphorothioate oligonucleotides as the basic active substances (Figure 7). In our opinion, the cumulative effect of the proposed antisense oligonucleotides combined with the special ointment formula will achieve a more pronounced apoptotic effect both in melanoma cell lines and on the skin of experimental animals. Ointments have a higher concentration of oil than creams. A higher concentration of oil in the preparation allows a higher rate of penetration of through the stratum corneum into the epidermis and dermis. This in turn allows the antisense oligonucleotides to reach the proposed zones of the melanoma precursor cells, which include dedifferentiated melanocytes, melanocyte progenitors in the bulge region of hair follicles, and neural crest-derived Schwann cell precursors [20,21,22,23]. Ointments also make it easier to treat the zones adjacent to the tumor focus, which is vital in aggressive cancers prone to metastasis. When examining the areas beyond the histologically recognizable boundaries of a melanoma, carcinogenic melanocytes (field cells) in the epidermis have been found up to 3 mm away [193]. Following genetic profiling, it was found that field cells represent an earlier phase of disease that precedes melanoma in situ. Nothing about the character of the tumor, including its depth or diameter, seems to correlate in any way with the extent to which field cells exist near it. This means that tumor depth is not a useful parameter with which to predict the extent of field cells. In addition, these results demonstrate that, on acral sites, melanoma field cells extend significantly into seemingly normal skin. The existence of these elusive field cells makes it easier to understand why certain types of melanoma tend to recur locally even after (apparent) complete excision [194] and explains why a significant number of patients diagnosed with intermediate-thickness (2–4 mm) cutaneous melanoma eventually suffer recurrence at local or distant sites [195]. It is not known what specific event initiates metastasis. One hypothesis is that metastasis occurs when cancer cells fuse with macrophages or other migratory bone marrow-derived cells [196]. This fusion would then activate the master regulatory genes that activate multiple pathways, particularly those related to epithelial–mesenchymal transition, such as SNAIL, SLUG, SPARC, and TWIST. In an effort to understand how this fusion could happen, spontaneous PADA melanoma was studied using electron microscopy. In this cancer, it was found that the melanin was located in autophagosomes, not in the cytoplasm, as is usually the case with melanocytes [197]. This demonstrates that cancer cells can acquire the phenotype of macrophages, lending support to the idea that this may be one method by which metastases may begin [198]. 

When investigating the potential of DNA insecticides, we conducted large-scale preparatory studies using topically applied antisense fragments of the IAP-Z gene [143,199,200] of the gypsy moth, *Lymantria dispar* L. At the larval stage, *L. dispar* cells, including epithelium cells, divide very actively (over a period of 2 months, the insect mass increases, on average, by 1000-fold), with a level of proliferation comparable to that of cancer cells. Experimental results have shown the possibility of using short 18–20-nucleotide-long antisense fragments to increase the rate of apoptotic processes in cells.

## 8. Conclusions

Deaths from melanoma and Merkel cell carcinoma happen most often after several surgical interventions. Therefore, there is time available during treatment that could be spent more efficiently. In our opinion, antisense oligonucleotides, which can be used in the form of targeted ointments or creams, provide particular hope for the elimination of cancer cells in the area near the tumor focus, both before and after surgery, to delay or even prevent the primary tumor from developing metastases and sending them to the lymph nodes. We also strongly believe that with the help of antisense techniques, Google searches for melanoma and Merkel cell carcinoma will be supplemented with the words ‘remission’ and even ‘complete cure’. In the meantime, there is hard work to be done. First, please examine your skin, because it is all a matter of timing when it comes to skin cancer, particularly melanoma and Merkel cell carcinoma. To determine whether any skin changes you find are likely to be skin cancer, ask your doctor to examine your skin as well.

## References

[1] Tsao H. (2001). Genetics of non-melanoma skin cancer. Arch. Dermatol..

[2] Diepgen T.L., Mahler V. (2002). The epidemiology of skin cancer. Br. J. Dermatol..

[3] Leiter U., Garbe C. (2008). Epidemiology of melanoma and non-melanoma skin cancer—The role of sunlight. Adv. Exp. Med. Biol..

[4] World Health Organization Skin Cancers /online/. http://www.who.int/uv/faq/skincancer/en/print.html.

[5] Apalla Z., Lallas A., Sotiriou E., Lazaridou E., Ioannides D. (2017). Epidemiological trends in skin cancer. Dermatol. Pract. Concept..

[6] Lomas A., Leonardi-Bee J., Bath-Hextall F. (2012). A systematic review of worldwide incidence of non-melanoma skin cancer. Br. J. Dermatol..

[7] Connolly S.M., Baker D.R., Coldiron B.M., Coldiron B.M., Fazio M.J., Storrs P.A., Vidimos A.T., Zalla M.J., Brewer J.D., Smith Begolka W. (2012). AAD/ACMS/ASDSA/ASMS 2012 appropriate use criteria for Mohs micrographic surgery: A report of the American Academy of Dermatology, American College of Mohs Surgery, American Society for Dermatologic Surgery Association, and the American Society for Mohs Surgery. J. Am. Acad. Dermatol..

[8] Karimkhani C., Green A.C., Nijsten T., Weinstock M.A., Dellavalle R.P., Naghavi M., Fitzmaurice C. (2017). The global burden of melanoma: Results from the Global Burden of Disease Study 2015. Br. J. Dermatol..

[9] Lemos B.D., Storer B.E., Iyer J.G., Phillips J.L., Bichakjian C.K., Fang L.C., Johnson T.M., Liegeois-Kwon N.J., Otley C.C., Paulson K.G. (2010). Pathologic nodal evaluation improves prognostic accuracy in Merkel cell carcinoma: Analysis of 5823 cases as the basis of the first consensus staging system. J. Am. Acad. Dermatol..

[10] Fitzgerald T.L., Dennis S., Kachare S.D., Vohra N.A., Wong J.H., Zervos E.E. (2015). Dramatic increase in the incidence and mortality from Merkel cell carcinoma in the United States. Am. Surg..

[11] Miller S.J., Alam M., Andersen J.S., Berg D., Bichakjian C.K., Bowen G.M., Cheney R.T., Glass L.F., Grekin R.C., Ho A.L. (2012). Dermatofibrosarcoma protuberans. J. Nat. Compr. Cancer Netw..

[12] Strassburg M.A. (1982). The global eradication of smallpox. Am. J. Infect. Control.

[13] Elwood J.M., Jopson J. (1997). Melanoma and sun exposure: An overview of published studies. Int. J. Cancer.

[14] Lowe N.J. (2006). An overview of ultraviolet radiation, sunscreens, and photo-induced dermatoses. Dermatol. Clin..

[15] Slominski A., Wortsman J. (2000). Neuroendocrinology of the skin. Endocr. Rev..

[16] Fuchs E. (2007). Scratching the surface of skin development. Nature.

[17] Slominski A.T., Zmijewski M.A., Skobowiat C., Zbytek B., Slominski R.M., Steketee J.D. (2012). Sensing the environment: Regulation of local and global homeostasis by the skin’s neuroendocrine system. Adv. Anat. Embryol. Cell Biol..

[18] Setlow R.B. (1974). The Wavelengths in Sunlight Effective in Producing Skin Cancer: A Theoretical Analysis. Proc. Natl. Acad. Sci. USA.

[19] Berwick M., Wiggins C. (2006). The current epidemiology of cutaneous malignant melanoma. Front. BioSci..

[20] Brash D.E., Heffernan T., Ngheim P., Wolff K., Goldsmith L.A., Katz S.I., Gilchrest B.A., Paller A.S., Leffell D.J. (2008). Carcinogenesis: Ultraviolet radiation. Fitzpatrick’s Dermatology in General Medicine.

[21] Donovan J. (2009). Review of the hair follicle origin hypothesis for basal cell carcinoma. Dermatol. Surg..

[22] Youssef K.K., Van Keymeulen A., Lapouge G., Beck B., Michaux C., Achouri Y., Sotiropoulou P.A., Blanpain C. (2010). Identification of the cell lineage at the origin of basal cell carcinoma. Nat. Cell Biol..

[23] Tlholoe M.M., Khammissa R.A., Bouckaert M., Altini M., Lemmer J., Feller L. (2015). Oral Mucosal Melanoma: Some Pathobiological Considerations and an Illustrative Report of a Case. Head Neck Pathol..

[24] Epstein E.H. (2008). Basal cell carcinomas: Attack of the hedgehog. Nat. Rev. Cancer.

[25] Peterson S.C., Eberl M., Vagnozzi A.N., Belkadi A., Veniaminova N.A., Verhaegen M.E., Bichakjian C.K., Ward N.L., Dlugosz A.A., Wong S.Y. (2015). Basal cell carcinoma preferentially arises from stem cells within hair follicle and mechanosensory niches. Cell Stem Cell..

[26] Visvader J.E. (2011). Cells of origin in cancer. Nature.

[27] Kim B.J., Kim H., Jin U.S., Minn K.W., Chang H. (2015). Wide local excision for dermatofibrosarcoma protuberans: A single-center series of 90 patients. BioMed Res. Int..

[28] Noujaim J., Thway K., Fisher C., Jones R.L. (2015). Dermatofibrosarcoma protuberans: From translocation to targeted therapy. Cancer Biol. Med..

[29] Becker J.C., zur Hausen A. (2014). Cells of origin in skin cancer. J. Investig. Dermatol..

[30] Schadendorf D., Lebbé C., Hausen zur A., Avril M.F., Hariharan S., Bharmal M., Becker J.C. (2017). Merkel cell carcinoma: Epidemiology, prognosis, therapy and unmet medical needs. Eur. J. Cancer.

[31] Arora R., Chang Y., Moore P.S. (2012). MCV and Merkel cell carcinoma: A molecular success story. Curr. Opin. Virol..

[32] Tilling T., Moll I. (2012). Which are the cells of origin in merkel cell carcinoma?. J. Skin Cancer.

[33] Wang T.S., Byrne P.J., Jacobs L.K., Taube J.M. (2011). Merkel cell carcinoma: Update and review. Semin. Cutan. Med. Surg..

[34] Young C. (2009). Solar ultraviolet radiation and skin cancer. Occup. Med. (Lond.).

[35] D’Orazio J., Jarrett S., Maro-Ortiz A., Scott T. (2013). UV Radiation and the skin. Int. J. Mol. Sci..

[36] Meeran S.M., Punathil T., Katiyar S.K. (2008). Interleukin-12-deficiency exacerbates inflammatory responses in UVirradiated skin and skin tumors. J. Investig. Dermatol..

[37] Rangwala S., Tsai K.Y. (2011). Roles of the immune system in skin cancer. Br. J. Dermatol..

[38] Benjamin C.L., Ananthaswamy H.N. (2007). p53 and the pathogenesis of skin cancer. Toxicol. Appl. Pharmacol..

[39] Narayanan D.L., Saladi R.N., Fox J.L. (2010). Ultraviolet radiation and skin cancer. Int. J. Dermatol..

[40] Liley J.B., McKenzie R.L. (2006). Where on Earth has the highest UV? UV Radiation and Its Effects: An Update. Natl. Inst. Water Atmos. Res..

[41] World Health Organization Ultraviolet Radiation (UV) /online/. www.who.int/uv/intersunprogramme/activities/uv_index/en/index3.html.

[42] Pleasance E.D., Cheetham R.K., Stephens P.J., McBride D.J., Humphray S.J., Greenman C.D., Varela I., Lin M.L., Ordóñez G.R., Bignell G.R. (2010). A comprehensive catalogue of somatic mutations from a human cancer genome. Nature.

[43] Elmets C.A., Ledet J.J., Athar M. (2014). Cyclooxygenases: Mediators of UV-induced skin cancer and potential targets for prevention. J. Investig. Dermatol..

[44] Timerman D., McEnery-Stonelake M., Joyce C.J., Nambudiri V.E., Hodi F.S., Claus E.B., Ibrahim N., Lin J.Y. (2017). Vitamin D deficiency is associated with a worse prognosis in metastatic melanoma. Oncotarget..

[45] Juzeniene A., Moan J. (2012). Beneficial effects of UV radiation other than via vitamin D production. Dermatoendocrinology.

[46] Feller L., Khammissa R.A., Kramer B., Altini M., Lemmer J. (2016). Basal cell carcinoma, squamous cell carcinoma and melanoma of the head and face. Head Face Med..

[47] Grossman D., Leffell D.J., Wolff K., Goldsmith L.A., Katz S.I., Gilchrest B.A., Paller A.S., Leffell D.J. (2008). Squamous cell carcinoma. Fitzpatrick’s Dermatology in General Medicine.

[48] Bakos L., Masiero N.C., Bakos R.M., Burttet R.M., Wagner M.B., Benzano D. (1979). European ancestry and cutaneous melanoma in Southern Brazil; I.K. Crombie Racial differences in melanoma incidence. Br. J. Cancer.

[49] Scherer D., Kumar R. (2010). Genetics of pigmentation in skin cancer–A review. Mutat. Res..

[50] Horrell E.M.W., Boulanger M., D’Orazio J.A. (2016). Melanocortin 1 receptor: Structure, function and regulation. Front. Genet..

[51] Grabowski J., Saltzstein S.L., Sadler G.R., Tahir Z., Blair S. (2008). A comparison of Merkel cell carcinoma and melanoma: Results from the california cancer registry. Clin. Med. Oncol..

[52] Mogha A., Fautrel A., Mouchet N., Guo N., Corre S., Adamski H., Watier E., Misery L., Galibert M.D. (2010). Merkel cell polyomavirus small T antigen mRNA level is increased following in vivo UV-radiation. PLoS ONE.

[53] Wong S.Q., Waldeck K., Vergara I.A., Schröder J., Madore J., Wilmott J.S., Colebatch A.J., De Paoli-Iseppi R., Li J., Lupat R. (2015). UV-associated mutations underlie the etiology of MCV-negative Merkel cell carcinomas. Cancer Res..

[54] Becker J.C., Stang A., DeCaprio J.A., Cerroni L., Lebbé C., Veness M., Nghiem P. (2017). Merkel cell carcinoma. Nat. Rev. Dis. Primers.

[55] Girschik J., Thorn K., Beer T.W., Heenan P.J., Fritschi L. (2011). Merkel cell carcinoma in Western Australia: A population-based study of incidence and survival. Br. J. Dermatol..

[56] Dabner M., McClure R.J., Harvey N.T., Budgeon C.A., Beer T.W., Amanuel B., Wood B.A. (2014). Merkel cell polyomavirus and p63 status in Merkel cell carcinoma by immunohistochemistry: Merkel cell polyomavirus positivity is inversely correlated with sun damage, but neither is correlated with outcome. Pathology.

[57] Chockalingam R., Downing C., Tyring S.K. (2015). Cutaneous squamous cell carcinomas in organ transplant recipients. J. Clin. Med..

[58] Heratizadeh A., Völker B., Kupsch E., Wichmann K., Kapp A., Werfel T. (2010). Successful symptomatic treatment of epidermodysplasia verruciformis with imiquimod 5% cream. Hautarzt.

[59] Moncrieff M. (2016). Excision margins for melanomas: How wide is enough?. Lancet Oncol..

[60] Di Trolio R., Simeone E., Di Lorenzo G., Buonerba C., Ascierto P.A. (2015). The use of interferon in melanoma patients: A systematic review. Cytokine Growth Factor Rev..

[61] Flaherty K.T., Puzanov I., Kim K.B., Ribas A., McArthur G.A., Sosman J.A., O’Dwyer P.J., Lee R.J., Grippo J.F., Nolop K. (2010). Inhibition of mutated, activated BRAF in metastatic melanoma. N. Eng. J. Med..

[62] Sosman J.A., Kim K.B., Schuchter L., Gonzalez R., Pavlick A.C., Weber J.S., McArthur G.A., Hutson T.E., Moschos S.J., Flaherty K.T. (2012). Survival in BRAF V600-mutant advanced melanoma treated with vemurafenib. N. Eng. J. Med..

[63] Nikolaou V.A., Stratigos A.J., Flaherty K.T., Tsao H. (2012). Melanoma: New insights and new therapies. J. Investig. Dermatol..

[64] Ji Z., Flaherty K.T., Tsao H. (2012). Targeting the RAS pathway in melanoma. Trends Mol. Med..

[65] Flaherty K.T. (2012). Targeting metastatic melanoma. Annu. Rev. Med..

[66] Hodi F.S., Oble D.A., Drappatz J., Velazquez E.F., Ramaiya N., Ramakrishna N., Day A.L., Kruse A., Mac Rae S., Hoos A. (2008). CTLA-4 blockade with ipilimumab induces significant clinical benefit in a female with melanoma metastases to the CNS. Nat. Clin. Pract. Oncol..

[67] Hodi F.S., O’Day S.J., McDermott D.F., Weber R.W., Sosman J.A., Haanen J.B., Gonzalez R., Robert C., Schadendorf D., Hassel J.C. (2010). Improved survival with ipilimumab in patients with metastatic melanoma. N. Eng. J. Med..

[68] Serrone L., Hersey P. (1999). The chemoresistance of human malignant melanoma: An update. Melanoma Res..

[69] Grossman D., Altieri D.C. (2001). Drug resistance in melanoma: Mechanisms, apoptosis, and new potential therapeutic targets. Cancer Metastasis Rev..

[70] Helmbach H., Rossmann E., Kern M.A., Schadendorf D. (2001). Drug-resistance in human melanoma. Int. J. Cancer.

[71] Glazer A.M., Winkelmann R.R., Farberg A.S., Rigel D.S. (2016). Analysis of trends in US melanoma incidence and mortality. JAMA Dermatol..

[72] Balch C.M., Buzaid A.C., Soong S.J., Atkins M.B., Cascinelli N., Coit D.G., Fleming I.D., Gershenwald J.E., Houghton A., Kirkwood J.M. (2001). Final version of the American Joint Committee on Cancer staging system for cutaneous melanoma. Clin. Oncol..

[73] Jansen B., Wacheck V., Heere-Ress E., Schlagbauer-Wadl H., Hoeller C., Lucas T., Hoermann M., Hollenstein U., Wolff K., Pehamberger H. (2000). Chemosensitisation of malignant melanoma by BCL2 antisense therapy. Lancet.

[74] Megahed M., Schon M., Selimovic D., Schon M.P. (2002). Reliability of diagnosis of melanoma in situ. Lancet.

[75] Zalaudek I., Hofmann-Wellenhof R., Cerroni L., Kerl H. (2002). “White” dysplastic melanocytic naevi. Lancet.

[76] Hudson L.E., Maithel S.K., Carlson G.W., Rizzo M., Murray D.R., Hestley A.C., Delman K.A. (2013). 1 or 2 cm margins of excision for T2 melanomas: Do they impact recurrence or survival?. Ann. Surg. Oncol..

[77] Ross M.I., Gershenwald J.E. (2011). Evidence-based treatment of early-stage melanoma. J. Surg. Oncol..

[78] Ross M.I., Balch C.M. (2016). Excision margins of melanoma make a difference: New data support an old paradigm. Ann. Surg. Oncol..

[79] Rees J.L. (2004). The genetics of sun sensitivity in humans. Am. J. Hum. Genet..

[80] Lv R., Sun Q. (2017). A Network Meta-Analysis of Non-Melanoma Skin Cancer (NMSC) Treatments: Efficacy and Safety Assessment. J. Cell. Biochem..

[81] Griffin L., Lear J. (2016). Photodynamic Therapy and Non-Melanoma Skin Cancer. Cancers.

[82] Cohen D., Lee P. (2016). Photodynamic Therapy for Non-Melanoma Skin Cancers. Cancers.

[83] Hamdan I., Donnelly R. (2017). Microneedle-assisted photodynamic therapy: Delivery of a NIR photosensitiser for the treatment of skin cancers. Photodiagnosis Photodyn. Ther..

[84] Cheraghi N., Cognetta A., Goldberg D. (2017). Radiation Therapy in Dermatology: Non-Melanoma Skin Cancer. J. Drugs Dermatol..

[85] Kumar R., Deep G., Agarwal R. (2015). An overview of ultraviolet B radiation-induced skin cancer chemoprevention by Silibinin. Curr. Pharmacol. Rep..

[86] Nahhas A.F., Scarbrough C.A., Trotter S.A. (2017). Review of the global guidelines on surgical margins for nonmelanoma skin cancers. J. Clin. Aesthetic Dermatol..

[87] Duprat J.P., Landman G., Salvajoli J.V., Brechtbühl E.R. (2011). A review of the epidemiology and treatment of Merkel cell carcinoma. Clinics.

[88] Allen P.J., Zhang Z.F., Coit D.G. (1999). Surgical management of Merkel cell carcinoma. Ann. Surg..

[89] Lemos B., Nghiem P. (2007). Merkel cell carcinoma: More deaths but still no pathway to blame. J. Investig. Dermatol..

[90] Mohs F. (1944). Chemosurgical treatment of cancer of the lip: A microscopically controlled method of excision. Arch. Surg..

[91] National Comprehensive Cancer Network NCCN Clinical Practice Guidelines in Oncology; Dermatofibrosarcoma Protuberans. http://www.nccn.org.

[92] McArthur G., Demetri G.D., Oosterom A., Heinrich M.C., Debiec-Rychter M., Corless C.L., Nikolova Z., Dimitrijevic S., Fletcher J.A. (2005). Molecular and clinical analysis of locally advanced dermatofibrosarcoma protuberans treated with imatinib: Imatinib target exploration consortium study B2225. J. Clin. Oncol..

[93] Bogucki B., Neuhaus I., Hurst E.A. (2012). Dermatofibrosarcoma protuberans: A review of the literature. Dermatol. Surg..

[94] Fields R.C., Hameed M., Qin L.X., Moraco N., Jia X., Maki R.G., Singer S., Brennan M.F. (2011). Dermatofibrosarcoma protuberans (DFSP): Predictors of recurrence and the use of systemic therapy. Ann. Surg. Oncol..

[95] Leigheb M., Zavattaro E., Bellinzona F., Furlan G., Leigheb G. (2010). Micrographic surgery (Tubingen torte technique) for the treatment of an invasive dermatofibrosarcoma protuberans with muscular involvement. G. Ital. Dermatol. Venereol..

[96] Madhunapantula S.V., Robertson G.P. (2012). Chemoprevention of melanoma. Adv. Pharmacol..

[97] Keith R.L. (2009). Chemoprevention of lung cancer. Proc. Am. Thorac. Soc..

[98] Surh Y.J. (2003). Cancer chemoprevention with dietary phytochemicals. Nat. Rev. Cancer.

[99] Benowitz S. (1996). Technology Motivating Industry. The Scientist.

[100] Jiang G., Li R.H., Sun C., Liu Y.Q., Zheng J.N. (2014). Dacarbazine combined targeted therapy versus dacarbazine alone in patients with malignant melanoma: a meta-analysis. PLoS ONE.

[101] Bajetta E., Del Vecchio M., Bernard-Marty C., Vitali M., Buzzoni R., Rixe O., Nova P., Aglione S., Taillibert S., Khayat D. (2002). Metastatic melanoma: Chemotherapy. Semin. Oncol..

[102] Serrone L., Zeuli M., Sega F.M., Cognetti F. (2000). Dacarbazine-based chemotherapy for metastatic melanoma: Thirty-year experience overview. J. Exp. Clin. Cancer Res..

[103] Avril M.F., Aamdal S., Grob J.J., Hauschild A., Mohr P., Bonerandi J.J., Weichenthal M., Neuber K., Bieber T., Gilde K. (2004). Fotemustine compared with dacarbazine in patients with disseminated malignant melanoma: A Phase III Study. J. Clin. Oncol..

[104] Chiarion Sileni V., Nortilli R., Aversa S.M., Paccagnella A., Medici M., Corti L., Favaretto A.G., Cetto G.L., Monfardini S. (2001). Phase II randomized study of dacarbazine, carmustine, cisplatin, and tamoxifen versus dacarbazine alone in advanced melanoma patients. Melanoma Res..

[105] Strickland L.R., Pal H.C., Elmets C.A., Afaq F. (2015). Targeting drivers of melanoma with synthetic small molecules and phytochemicals. Cancer Lett..

[106] Richtig G., Hoeller C., Kashofer K., Aigelsreiter A., Heinemann A., Kwong L.N., Pichler M., Richtig E. (2017). Beyond the BRAFV600E hotspot-biology and clinical implications of rare BRAF gene mutations in melanoma patients. Br. J. Dermatol..

[107] Edwards R.H., Ward M.R., Wu H., Medina C.A., Brose M.S., Volpe P., Nussen-Lee S., Haupt H.M., Martin A.M., Herlyn M. (2004). Absence of BRAF mutations in UV-protected mucosal melanomas. J. Med. Genet..

[108] Ascierto P.A., Kirkwood J.M., Grob J.J., Simeone E., Grimaldi A.M., Maio M., Palmieri G., Testori A., Marincola F.M., Mozzillo N. (2012). The role of BRAF V600 mutation in melanoma. J. Transl. Med..

[109] Mukherjee N., Schwan J.V., Fujita M., Norris D.A., Shellman Y.G. (2015). Alternative treatments for melanoma: Targeting BCL-2 family members to de-bulk and kill cancer stem cells. J. Investig. Dermatol..

[110] Liu F., Cao J., Wu J., Sullivan K., Shen J., Ryu B., Xu Z., Wei W., Cui R. (2013). Stat3-targeted therapies overcome the acquired resistance to vemurafenib in melanomas. J. Investig. Dermatol..

[111] Becker T.M., Boyd S.C., Mijatov B., Gowrishankar K., Snoyman S., Pupo G.M., Scolyer R.A., Mann G.J., Kefford R.F., Zhang X.D. (2014). Mutant B-RAF-Mcl-1 survival signaling depends on the STAT3 transcription factor. Oncogene.

[112] Dong H., Strome S.E., Salomao D.R., Tamura H., Hirano F., Flies D.B., Roche P.C., Lu J., Zhu G., Tamada K. (2002). Tumor-associated B7-H1 promotes T-cell apoptosis: A potential mechanism of immune evasion. Nat. Med..

[113] Marconcini R., Spagnolo F., Stucci L.S., Ribero S., Marra E., Rosa F., Picasso V., Di Guardo L., Cimminiello C., Cavalieri S. (2018). Current status and perspectives in immunotherapy for metastatic melanoma. Oncotarget.

[114] Munoz-Couselo E., Adelantado E.Z., Ortiz C., Garcia J.S., Perez-Garcia J. (2017). NRAS-mutant melanoma: Current challenges and future prospect. OncoTargets Ther..

[115] Jakob J.A., Bassett R.L., Ng C.S., Curry J.L., Joseph R.W., Alvarado G.C., Rohlfs M.L., Richard J., Gershenwald J.E., Kim K.B. (2012). NRAS mutation status is an independent prognostic factor in metastatic melanoma. Cancer.

[116] Curtin J.A., Fridlyand J., Kageshita T., Patel H.N., Busam K.J., Kutzner H., Cho K.H., Aiba S., Bröcker E.B., LeBoit P.E. (2005). Distinct sets of genetic alterations in melanoma. N. Engl. J. Med..

[117] Lee J.H., Choi J.W., Kim Y.S. (2011). Frequencies of BRAF and NRAS mutations are different in histological types and sites of origin of cutaneous melanoma: A meta-analysis. Br. J. Dermatol..

[118] Erstad D.J., Cusack J.C. (2014). Mutational analysis of Merkel cell carcinoma. Cancers.

[119] Larramendy M.L., Koljonen V., Bohling T., Tukiainen E., Knuutila S. (2004). Recurrent DNA copy number changes revealed by comparative genomic hybridization in primary Merkel cell carcinomas. Mod. Pathol..

[120] Xie H., Lee L., Caramuta S., Hoog A., Browaldh N., Bjornhagen V., Larsson C., Lui W.O. (2014). MicroRNA expression patterns related to Merkel cell polyomavirus infection in human Merkel cell carcinoma. J. Investig. Dermatol..

[121] Engels E.A., Frisch M., Goedert J.J., Biggar R.J., Miller R.W. (2002). Merkel cell carcinoma and HIV infection. Lancet.

[122] Busam K.J., Jungbluth A.A., Rekthman N., Coit D., Pulitzer M., Bini J., Arora R., Hanson N.C., Tassello J.A., Frosina D. (2009). Merkel cell polyomavirus expression in Merkel cell carcinomas and its absence in combined tumors and pulmonary neuroendocrine carcinomas. Am. J. Surg. Pathol..

[123] Tadmor T., Aviv A., Polliack A. (2011). Merkel cell carcinoma, chronic lymphocytic leukemia and other lymphoproliferative disorders: An old bond with possible new viral ties. Ann. Oncol..

[124] Laude H.C., Jonchere B., Maubec E., Carlotti A., Marinho E., Couturaud B., Peter M., Sastre-Garau X., Avril M.F., Dupin N. (2010). Distinct merkel cell polyomavirus molecular features in tumour and non tumour specimens from patients with merkel cell carcinoma. PLoS Pathog..

[125] Sahi H., Savola S., Sihto H., Koljonen V., Bohling T., Knuutila S. (2014). Rb1 gene in merkel cell carcinoma: Hypermethylation in all tumors and concurrent heterozygous deletions in the polyomavirus-negative subgroup. APMIS.

[126] Popp S., Waltering S., Herbst C., Moll I., Boukamp P. (2002). Uv-b-type mutations and chromosomal imbalances indicate common pathways for the development of merkel and skin squamous cell carcinomas. Int. J. Cancer.

[127] Cohen P.R., Tomson B.N., Elkin S.K., Marchlik E., Carter J.L., Kurzrock R. (2016). Genomic portfolio of Merkel cell carcinoma as determined by comprehensive genomic profiling: Implications for targeted therapeutics. Oncotarget..

[128] Feng H., Shuda M., Chang Y., Moore P.S. (2008). Clonal integration of a polyomavirus in human merkel cell carcinoma. Science.

[129] Wong H.H., Wang J. (2009). Epstein-Barr virus positive diffuse large B-cell lymphoma of the elderly. Leuk. Lymphoma.

[130] Heath M., Jaimes N., Lemos B., Mostaghimi A., Wang L.C., Penas P.F., Nghiem P. (2008). Clinical characteristics of merkel cell carcinoma at diagnosis in 195 patients: The aeiou features. J. Am. Acad. Dermatol..

[131] Schwartz J.L., Wong S.L., McLean S.A., Hayman J.A., Lao C.D., Kozlow J.H., Malloy K.M., Bradford C.R., Frohm M.L., Fullen D.R. (2014). NCCN guidelines implementation in the multidisciplinary Merkel cell carcinoma program at the University of Michigan. J. Natl. Compr. Cancer Netw..

[132] Raju S., Vazirnia A., Totri C., Hata T.R. (2014). Treatment of Merkel cell carcinoma of the head and neck: A systematic review. Dermatol. Surg..

[133] Prewett S.L., Ajithkumar T. (2015). Merkel cell carcinoma: Current management and controversies. Clin. Oncol..

[134] Munoz I.P., Masferrer J.P., Vegas J.O., Montalvo M.S.M., Diaz R.J., Casas A.M.P. (2013). Merkel cell carcinoma from 2008 to 2012: Reaching a new level of understanding. Cancer Treat. Rev..

[135] Nghiem P., Bhatia S., Daud A., Friedlander P., Kluger H., Kohrt H., Kudchadkar R., Lipson E., Lundgren L., Margolin K. (2015). Activity of PD-1 blockade with pembrolizumab as first systemic therapy in patients with advanced Merkel cell carcinoma [Abstract 22LBA: In Proceedings of the 18th ECCO-40th ESMA European Cancer Congress, Vienna, Austria, 25–29 September 2015]. Eur. J. Cancer.

[136] Moshiri A.S., Nghiem P. (2014). Milestones in the staging, classification, and biology of Merkel cell carcinoma. J. Natl. Compr. Canc. Netw..

[137] Miller N.J., Bhatia S., Parvathaneni U., Iyer J.G., Nghiem P. (2013). Emerging and mechanism-based therapies for recurrent or metastatic Merkel cell carcinoma. Curr. Treat. Options Oncol..

[138] Aldabagh B., Joo J., Yu S.S. (2014). Merkel cell carcinoma: Current status of targeted and future potential for immunotherapies. Semin. Cutan. Med. Surg..

[139] Tothill R., Estall V., Rischin D. (2015). Merkel cell carcinoma: Emerging biology, current approaches, and future directions. Am. Soc. Clin. Oncol. Educ. Book.

[140] Wong E., Goldberg T. (2014). Mipomersen (kynamro): A novel antisense oligonucleotide inhibitor for the management of homozygous familial hypercholesterolemia. Pharm. Ther..

[141] Stein A., Castanotto D. (2017). FDA-Approved Oligonucleotide Therapies in 2017. Mol. Ther..

[142] Lim K.R., Maruyama R., Yokota T. (2017). Eteplirsen in the treatment of Duchenne muscular dystrophy. Drug Des. Dev. Ther..

[143] Oberemok V.V., Laikova K.V., Repetskaya A.I., Kenyo I.M., Gorlov M.V., Kasich I.N., Krasnodubets A.M., Gal’chinsky N.V., Fomochkina I.I., Zaitsev A.S. (2018). A Half-Century History of Applications of Antisense Oligonucleotides in Medicine, Agriculture and Forestry: We Should Continue the Journey. Molecules.

[144] Tarhini A.A., Kirkwood J.M. (2007). Oblimersen in the treatment of metastatic melanoma. Future Oncol..

[145] De Smet M.D., Meenken C.J., van den Horn G.J. (1999). Fomivirsen: A phosphorothioate oligonucleotide for the treatment of CMV retinitis. Ocul. Immunol. Inflamm..

[146] Moreno P.M., Pego A.P. (2014). Therapeutic antisense oligonucleotides against cancer: Hurdling to the clinic. Front. Chem..

[147] Fisher D.E. (1994). Apoptosis in cancer therapy: Crossing the threshold. Cell.

[148] Olie R.A., Hafner C., Kuttel R., Sigrist B., Willers J., Dummer R., Hall J., Stahel R.A., Zangemeister-Wittke U. (2002). BCL-2 and BCL-XL antisense oligonucleotides induce apoptosis in melanoma cells of different clinical stages. J. Investig. Dermatol..

[149] Soengas M.S., Lowe S.W. (2003). Apoptosis and melanoma chemoresistance. Oncogene.

[150] Bedikian A.Y., Millward M., Pehamberger H., Conry R., Gore M., Trefzer U., Pavlick A.C., DeConti R., Hersh E.M., Hersey P. (2006). BCL-2 antisense (oblimersen sodium) plus dacarbazine in patients with advanced melanoma: The oblimersen melanoma study group. J. Clin. Oncol..

[151] Luo X., Budihardjo I., Zou H., Slaughter C., Wang X. (1998). Bid, a Bcl2 interacting protein, mediates cytochrome c release from mitochondria in response to activation of cell surface death receptors. Cell.

[152] Vogler M., Dinsdale D., Dyer M.J., Cohen G.M. (2009). BCL-2 inhibitors: Small molecules with a big impact on cancer therapy. Cell. Death Differ..

[153] Jansen B., Schlagbauer-Wadl H., Brown B.D., Bryan R.N., van Elsas A., Müller M., Wolff K., Eichler H.G., Pehamberger H. (1998). BCL-2 antisense therapy chemosensitizes human melanoma in SCID mice. Nat. Med..

[154] Klasa R.J., Gillum A.M., Klem R.E., Frankel S.R. (2002). Oblimersen BCL-2 antisense: Facilitating apoptosis in anticancer treatment. Antisense Nucleic Acid Drug Dev..

[155] Cheson B.D. (2007). Oblimersen for the treatment of patients with chronic lymphocytic leukemia. Ther. Clin. Risk Manag..

[156] Mita M.M., Ochoa L., Rowinsky E.K., Kuhn J., Schwartz G., Hammond L.A., Patnaik A., Yeh I.-T., Izbicka E., Berg K. (2006). A phase I, pharmacokinetic and biologic correlative study of oblimersen sodium (Genasense™, G3139) and irinotecan in patients with metastatic colorectal cancer. Ann. Oncol..

[157] Houben R., Schrama D., Becker J.C. (2009). Molecular pathogenesis of Merkel cell carcinoma. Exp. Dermatol..

[158] Ambrosini G., Adida C., Altieri D.C. (1997). A novel anti-apoptosis gene, survivin, expressed in cancer and lymphoma. Nat. Med..

[159] Grossman D., McNiff J.M., Li F., Altieri D.C. (1999). Expression and targeting of the apoptosis inhibitor, survivin, in human melanoma. J. Investig. Dermatol..

[160] Marusawa H., Matsuzawa S., Welsh K., Zou H., Armstrong R., Tamm I., Reed J.C. (2003). HBXIP functions as a cofactor of survivin in apoptosis suppression. EMBO J..

[161] Cory S., Adams J.M. (2002). The Bcl2 family: Regulators of the cellular life-or-death switch. Nat. Rev. Cancer.

[162] Pennati M., Folini M., Zaffaroni N. (2007). Targeting survivin in cancer therapy: Fulfilled promises and open questions. Carcinogenesis.

[163] Talbot D.C., Ranson M., Davies J., Lahn M., Callies S., Andre V., Kadam S., Burgess M., Slapak C., Olsen A.L. (2010). Tumor survivin is downregulated by the antisense oligonucleotide LY2181308: A proof-of-concept, first-in-human dose study. Clin. Cancer Res..

[164] Ryan B.M., O’Donovan N., Duffy M.J. (2009). Survivin: A new target for anti-cancer therapy. Cancer Treat. Rev..

[165] Herrington W.G., Talbot D.C., Lahn M.M., Brandt J.T., Callies S., Nagle R., Winearls C.G., Roberts I.S. (2011). Association of long-term administration of the survivin mRNA-targeted antisense oligonucleotide LY2181308 with reversible kidney injury in a patient with metastatic melanoma. Am. J. Kidney Dis..

[166] Feinmesser M., Halpern M., Fenig E., Tsabari C., Hodak E., Sulkes J., Brenner B., Okon E. (1999). Expression of the apoptosis-related oncogenes BCL-2, bax, and p53 in Merkel cell carcinoma. Can they predict treatment response and clinical outcome?. Hum. Pathol..

[167] Kennedy M.M., Blessing K., King G., Kerr K.M. (1996). Expression of BCL-2 and p53 in Merkel cell carcinoma. An immunohistochemical study. Am. J. Dermatopathol..

[168] Schlagbauer-Wadl H., Klosner G., Heere-Ress E., Waltering S., Moll I., Wolff K., Pehamberger H., Jansen B. (2000). BCL-2 antisense oligonucleotides (G3139) inhibit Merkel cell carcinoma growth in SCID mice. J. Investig. Dermatol..

[169] Shah M.H., Varker K.A., Collamore M., Zwiebel J.A., Coit D., Kelsen D., Chung K.Y. (2007). G3139 therapy (Genasense) in patients with advanced merkel cell carcinoma. Proc. Am. Assoc. Cancer Res. (AACR).

[170] Stein C.A. (1995). Does antisense exist?. Nature Med..

[171] Wraight C.J., White P.J. (2001). Antisense oligonucleotides in cutaneous therapy. Pharmacol. Ther..

[172] Dokka S., Cooper S.R., Kelly S., Hardee G.E., Karras J.G. (2005). Dermal delivery of topically applied oligonucleotides via follicular transport in mouse skin. J. Investig. Dermatol..

[173] Mehta R.C., Stecker K.K., Cooper S.R., Templin M.V., Tsai Y.J., Condon T.P., Bennett C.F., Hardee G.E. (2000). Intercellular adhesion molecule-1 suppression in skin by topical delivery of antisense oligonucleotides. J. Investig. Dermatol..

[174] Yacyshyn B.R., Bowen-Yacyshyn M.B., Jewell L., Tami J.A., Bennett C.F., Kisner D.L., Shanahan W.R. (1998). A placebo-controlled trial of ICAM-1 antisense oligonucleotide in the treatment of Crohn’s disease. Gastroenterology.

[175] Beltinger C., Saragovi H.U., Smith R.M., LeSauteur L., Shah N., DeDionisio L., Christensen L., Raible L., Jarett L., Gewirtz A.M. (1995). Binding uptake and intracellular trafficking of phosphorothioate modified oligodeoxynucleotides. J. Clin. Investig..

[176] Leonetti C., Biroccio A., Benassi B., Stringaro A., Stoppacciaro A., Semple S.C., Zupi G. (2001). Encapsulation of c-myc antisense oligodeoxynucleotides in lipid particles improves antitumoral efficacy in vivo in a human melanoma line. Cancer Gene Ther..

[177] Fattal E., Bochot A. (2008). State of the art and perspectives for the delivery of antisense oligonucleotides and siRNA by polymeric nanocarriers. Int. J. Pharm..

[178] Brand R.M., Iversen P.L. (2000). Transdermal delivery of antisense compounds. Adv. Drug Deliv. Rev..

[179] Arora V., Hannah T.L., Iversen P.L., Brand R.M. (2002). Transdermal use of phospharadiamidate morpholino oligomer AVI-4472 inhibits cytochrome P450 3A2 activity in male rats. Pharm. Res..

[180] Hsu T., Mitragotri S. (2011). Delivery of siRNA and other macromolecules into skin and cells using a peptide enhancer. Proc. Natl. Acad. Sci. USA.

[181] Regnier V., Morre N.D., Jadoul A., Preat V. (1999). Mechanism of a phosphorothioate oligonucleotide delivery by skin electroporation. Int. J. Pharm..

[182] Tezel A., Dokka S., Kelly S., Hardee G.E., Mitragotri S. (2004). Topical delivery of anti-sense oligonucleotides using low frequency sonophoresis. Pharm. Res..

[183] Lee W.R., Shen S.C., Liu C.R., Fang C.L., Hu C.H., Fang J.Y. (2006). Erbium:YAG laser-mediated oligonucleotide and DNA delivery via the skin: An animal study. J. Control. Release.

[184] Lin W., Cormier M., Samiee A., Griffin A., Johnson B., Teng C.L., Hardee G.E., Daddona P.E. (2001). Transdermal delivery of antisense oligonucleotides with microprojection patch (MacrofluxR) technology. Pharm. Res..

[185] Venuganti V.V., Saraswathy M., Dwivedi C., Kaushik R.S., Perumal O.P. (2015). Topical gene silencing by iontophoretic delivery of an antisense oligonucleotide-dendrimer nanocomplex: The proof of concept in a skin cancer mouse model. Nanoscale.

[186] Siu K.S., Chen D., Zheng X., Zhang X., Johnston N., Liu Y., Yuan K., Koropatnick J., Gillies E.R., Min W.P. (2014). Non-covalently functionalized single-walled carbon nanotube for topical siRNA delivery into melanoma. Biomaterials.

[187] Kirschner N., Rosenthal R., Furuse M., Moll I., Fromm M., Brandner J.M. (2013). Contribution of Tight Junction Proteins to Ion, Macromolecule, and Water Barrier in Keratinocytes. J. Investig. Dermatol..

[188] Zakrewsky M., Kumar S., Mitragotri S. (2015). Nucleic acid delivery into skin for the treatment of skin disease: Proofs-of-concept, potential impact, and remaining challenges. J. Control. Release.

[189] Regnier V., Le Doan T., Preat V. (1998). Parameters controlling topical delivery of oligonucleotides by electroporation. J. Drug. Target..

[190] White P.J., Gray A.C., Fogarty R.D., Sinclair R.D., Thumiger S.P., Werther G.A., Wraight C.J. (2002). C-5 propyne-modified oligonucleotides penetrate the epidermis in psoriatic and not normal human skin after topical application. J. Investig. Dermatol..

[191] Li L., Lishko V., Hoffman R.M. (1993). Liposome targeting of high molecular weight DNA to the hair follicles of histocultured skin: A model for gene therapy of the hair growth processes. In Vitro Cell. Dev. Biol. Anim..

[192] Lieb L.M., Liimatta A.P., Bryan R.N., Brown B.D., Krueger G.G. (1997). Description of the intrafollicular delivery of large molecular weight molecules to follicles of human scalp skin in vitro. J. Pharm. Sci..

[193] Bastian B.C., Kashani-Sabet M., Hamm H., Godfrey T., Moore D.H., Bröcker E.B., LeBoit P.E., Pinkel D. (2000). Gene amplifications characterize acral melanoma and permit the detection of occult tumor cells in the surrounding skin. Cancer Res..

[194] North J.P., Kageshita T., Pinkel D., LeBoit P.E., Bastian B.C. (2008). Distribution and significance of occult intraepidermal tumor cells surrounding primary melanoma. J. Invest. Dermatol..

[195] Chin L., Garraway L.A., Fisher D.E. (2006). Malignant melanoma: Genetics and therapeutics in the genomic era. Genes Dev..

[196] Pawelek J.M., Chakraborty A.K. (2008). Fusion of tumour cells with bone marrow-derived cells: A unifying explanation for metastasis. Nat. Rev. Cancer.

[197] Pawelek J.M., Chakraborty A.K., Rachkovsky M.L., Orlow S.J., Bolognia J.L., Sodi S.A. (1999). Altered N-glycosylation in macrophage x melanoma fusion hybrids. Cell. Mol. Biol. (Noisy-le-grand).

[198] Zbytek B., Carlson J.A., Granese J., Ross J., Mihm M.C., Slominski A. (2008). Current concepts of metastasis in melanoma. Expert Rev. Dermatol..

[199] Oberemok V.V., Laikova K.V., Zaitsev A.S., Shumskykh M.N., Kasich I.N., Gal’chinsky N.V., Bekirova V.V., Makarov V.V., Agranovsky A.A., Gushchin V.A. (2017). Molecular Alliance of Lymantria dispar Multiple Nucleopolyhedrovirus and a Short Unmodified Antisense Oligonucleotide of Its Anti-Apoptotic IAP-3 Gene: A Novel Approach for Gypsy Moth Control. Int. J. Mol. Sci..

[200] Oberemok V.V., Laikova K.V., Zaitsev A.S., Nyadar P.M., Gninenko Y.I., Gushchin V.A., Makarov V.V., Agranovsky A.A. (2017). Topical treatment of LdMNPV-infected gypsy moth caterpillars with 18 nucleotides long antisense fragment from LdMNPV IAP-3 gene triggers higher level of apoptosis in the infected cells and mortality of the pest. J. Plant Prot. Res..

